# Molecular Identification of Collagen 17a1 as a Major Genetic Modifier of Laminin Gamma 2 Mutation-Induced Junctional Epidermolysis Bullosa in Mice

**DOI:** 10.1371/journal.pgen.1004068

**Published:** 2014-02-13

**Authors:** Thomas J. Sproule, Jason A. Bubier, Fiorella C. Grandi, Victor Z. Sun, Vivek M. Philip, Caroline G. McPhee, Elisabeth B. Adkins, John P. Sundberg, Derry C. Roopenian

**Affiliations:** 1 The Jackson Laboratory, Bar Harbor, Maine, United States of America; 2 Genetics Program, Sackler School of Graduate Biomedical Sciences, Tufts University, Boston, Massachusetts, United States of America; Stanford University School of Medicine, United States of America

## Abstract

Epidermolysis Bullosa (EB) encompasses a spectrum of mechanobullous disorders caused by rare mutations that result in structural weakening of the skin and mucous membranes. While gene mutated and types of mutations present are broadly predictive of the range of disease to be expected, a remarkable amount of phenotypic variability remains unaccounted for in all but the most deleterious cases. This unexplained variance raises the possibility of genetic modifier effects. We tested this hypothesis using a mouse model that recapitulates a non-Herlitz form of junctional EB (JEB) owing to the hypomorphic *jeb* allele of laminin gamma 2 (*Lamc2*). By varying normally asymptomatic background genetics, we document the potent impact of genetic modifiers on the strength of dermal-epidermal adhesion and on the clinical severity of JEB in the context of the *Lamc2^jeb^* mutation. Through an unbiased genetic approach involving a combination of QTL mapping and positional cloning, we demonstrate that *Col17a1* is a strong genetic modifier of the non-Herlitz JEB that develops in *Lamc2^jeb^* mice. This modifier is defined by variations in 1–3 neighboring amino acids in the non-collagenous 4 domain of the collagen XVII protein. These allelic variants alter the strength of dermal-epidermal adhesion in the context of the *Lamc2^jeb^* mutation and, consequentially, broadly impact the clinical severity of JEB. Overall the results provide an explanation for how normally innocuous allelic variants can act epistatically with a disease causing mutation to impact the severity of a rare, heritable mechanobullous disorder.

## Author Summary

Epidermolysis bullosa (EB) is a group of rare genetic Mendelian disorders that result in mechanical fragility of the skin and mucosal membranes. Junctional EB is a subset caused by mutations that result in cleavage of the dermal-epidermal junction. All forms of EB demonstrate substantial variability in their clinical phenotype that is not readily explained. The possibility of genetic modifiers as the cause of this variability has been difficult to address in humans. We apply a mouse model carrying a hypomorphic allele of the laminin gamma 2 (*Lamc2*) gene to address the possibility of genetic modifiers of JEB. We document the potent impact of differing genetic backgrounds on multiple facets of the JEB syndrome expressed in these mice and show that three neighboring amino acid changes within the non-collagenous domain 4 of the collagen XVII protein strongly modify their disease. The study provides a molecular explanation of how a primary mutation that weakens one component of the cutaneous basement membrane is influenced by normally innocuous allelic variants of another component to affect strength of dermal-epidermal adhesion and consequently, the severity of JEB. This approach may guide the genetic prognosis and diagnosis of human EB disorders.

## Introduction

Epidermolysis bullosa (EB) is a group of rare heritable disorders that result in mechanical fragility of the skin and mucosal membranes. These disorders are caused by defects in any one of at least 18 genes whose encoded proteins control the integrity of the dermal and epidermal layers [1]–[5]. Depending upon where the tissue separation occurs, EB can be subdivided into three main groups: EB simplex, in which blister formation leads to the disruption of the basal keratinocytes; junctional EB (JEB), in which tissue cleavage occurs in the lamina lucida of the cutaneous basement membrane (BM); and dystrophic EB, in which cleavage occurs beneath the lamina densa of the dermal-epidermal BM zone [6]–[9]. A fourth type, Kindler syndrome, is characterized by cleavage within and beneath the basement membrane zone [6], [10].

JEB typically results from mutations in any one of the three genes encoding subunits of laminin 332, a heterotrimeric macromolecule comprised of laminin α3 (*LAMA3*), laminin β3 (*LAMB3*), and laminin γ2 (*LAMC2*); in either integrin α6 (*ITGA6*) or β4 (*ITGB4*), which form the α6β4 integrin heterodimer; or in *COL17A1*, which forms the collagen XVII homotrimer [6], [11]–[13]. The more severe Herlitz type (JEB-H), which commonly results in death during infancy or early childhood, is usually characterized by recessively acting loss-of-function mutations leading to premature termination codons (PTCs) in any one of the three subunit genes encoding the laminin 332 protein complex. Non-Herlitz JEB (JEB-nH) presents more moderate phenotypes, usually with much longer patient survival, often not displaying overt symptoms until adolescence or adulthood. JEB-nH results from less severe mutations (missense; in-frame splicing) of the laminin 332 genes, or mutations ranging from missense to loss-of-function in *COL17A1* and can be subcategorized based on generalized or localized manifestations as well as their clinical presentations [3], [6], [13]–[15]. JEB-PA is in many ways phenotypically similar to JEB-nH but is caused by mutations in *ITGA6* or *ITGB4* and is classified separately based on the additional phenotype of pyloric atresia [6].

While a unifying feature of all subforms of EB is adherence to simple Mendelian patterns of inheritance, considerable phenotypic variation in disease severity can persist even within subtypes of EB and within affected families. Genetic modifiers may account for this incomplete penetrance and the “vastly different” clinical phenotypes that are commonly observed [1], [13], [15]–[19]. Direct investigation into potential genetic modifiers of EB in humans is extremely difficult due to the rarity of these disorders and the challenges of acquiring comparable data from dispersed patients and physicians. Here we utilize a mouse model to investigate the possibility of genetic modifiers of JEB. Mice homozygous for the hypomorphic *jeb* allele of one component of the laminin 332 complex, *Lamc2*, develop a simple autosomal recessive Mendelian disorder with a spectrum of abnormalities that are a remarkable phenocopy of the cutaneous and non-cutaneous features of non-Herlitz JEB [20]. Here, we use an unbiased genetic approach to document the potent impact of differing genetic backgrounds on multiple facets of the JEB syndrome expressed in these mice. Quantitative trait loci (QTL) analysis and recombination mapping lead to the identification of collagen XVII (*Col17a1*) as a major genetic modifier of JEB in *Lamc2^jeb^* mice. Ultrafine recombination mapping limited the causal variations to three neighboring amino acid changes within the non-collagenous domain 4 (NC4) of this protein. This identification of a genetic modifier provides a molecular explanation of how epistasis between a primary mutation that weakens one component of the cutaneous basement membrane, laminin 332, and normally innocuous allelic variants of another component, collagen XVII, affects the strength of dermal-epidermal adhesion and consequently modifies the severity of JEB.

## Results

### Strain background causes substantial variation in the onset and severity of JEB-nH caused by the *Lamc2^jeb^* mutation

**Figure 1:**
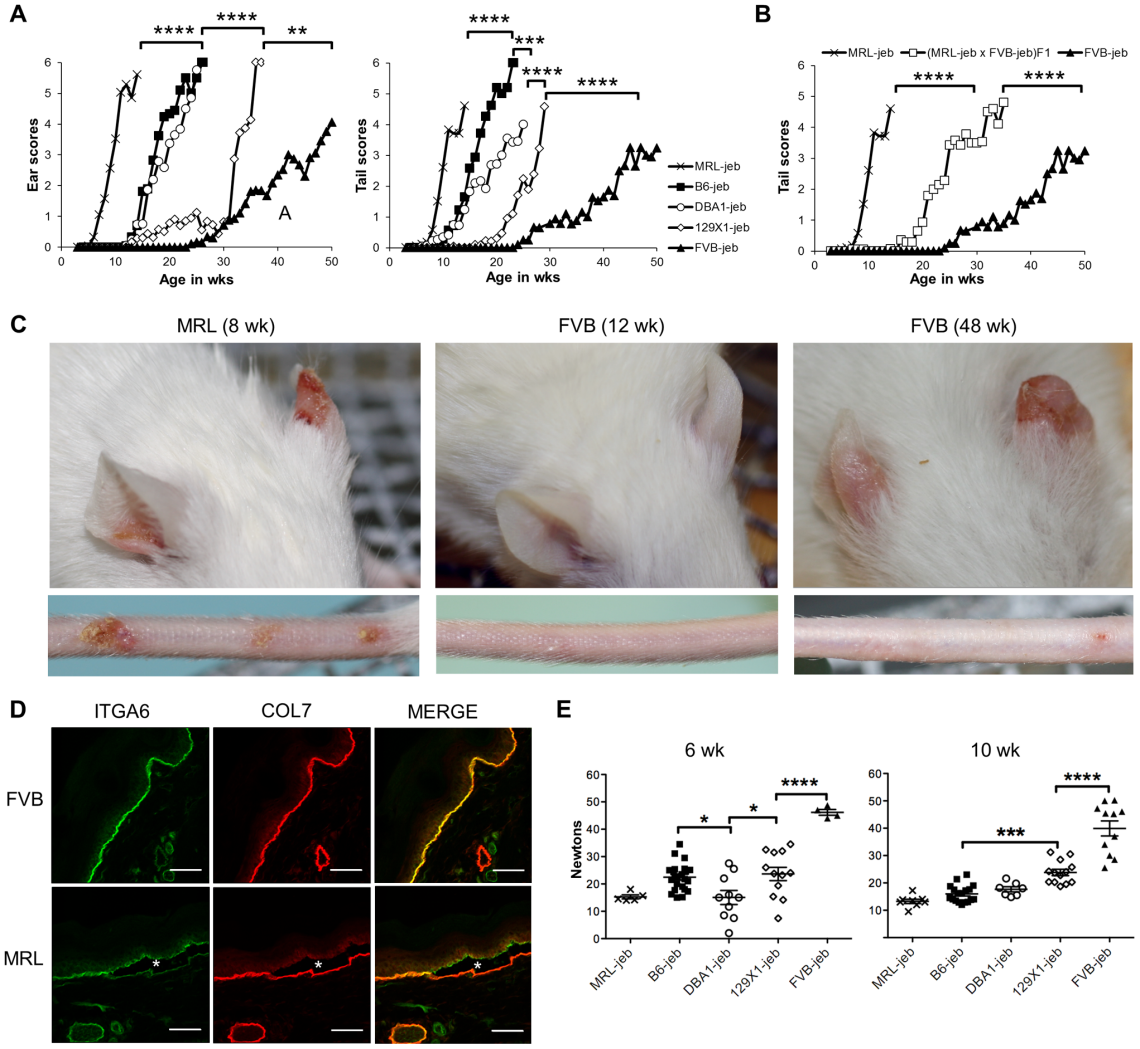
Mouse strain background causes substantial variation in the onset and severity of JEB-nH in *Lamc2^jeb/jeb^* mice. **A**, Average ear and tail scores from 0 (unaffected) to 6 (severe) demonstrate a range of ages of onset. N = 4–50 mice per strain per timepoint. **B**, Average tail scores for (MRL × FVB) F_1_ mice are intermediate compared to the parental strains. N = 5–16 F_1_ mice per timepoint. **C**, Representative ear and tail images of MRL at 8 wks and FVB at 12 and 48 wks demonstrate the typical early onset MRL and late onset FVB phenotypes. **D**, Representitive fluorescent imaging of cross-section of tail skin of 7–8 week old male mice demonstrates tissue separation (*) at the lamina lucida in MRL which does not occur in age-matched FVB mice. **E**, Differential tail tension measurements (in Newtons) in mice 6 and 10 weeks of age for the same strains as (A). (datapoints are measurements for individual mice). All mice are male *Lamc2^jeb^* ^/j*eb*^ homozygotes. *, *P*≤0.05; ***; ≤0.001; ****, ≤0.0001.

129X1/SvJ mice homozygous for the hypomorphic *Lamc2^jeb^* mutation develop a progressive form of JEB characterized most obviously by blistering and scarring of the ears and tails as the result of separations within the dermal-epidermal BM [20]. To address whether modifier genes influence this syndrome, a survey of mouse strain background effects was conducted. The *Lamc2^jeb^* allele arose on strain 129X1/SvJ and was backcrossed 10 generations onto four other strain backgrounds: C57BL/6J (B6), DBA/1J (DBA1), FVB/NJ (FVB), and MRL/MpJ (MRL). Cohorts of *Lamc2^jeb/jeb^* males from each strain were aged, visually inspected weekly, and quantitatively scored for the severity of ear and tail lesions (Figure 1A, C). Striking differences in the age of onset and the intensity of these lesions among the strains were observed, with the MRL background showing the earliest onset, followed by B6 and DBA1, 129X1, and with FVB being most resistant. Similar strain patterns were observed for females, though with delayed onset and less susceptibility to tail lesions (Figure S1A). To determine the inheritance patterns of susceptibility and resistance, we analyzed *Lamc2^jeb/jeb^* progeny from an F1 cross of the most susceptible (MRL) and most resistant (FVB) strain backgrounds. These results were consistent with co-dominant inheritance (Figure 1B). As would be expected, immunofluorescence imaging of tail skin confirmed separation at the plane of the lamina lucida in 7–8 wk old male MRL-*Lamc2^jeb/jeb^* mice, which was not apparent at this age in FVB-*Lamc2^jeb/jeb^* mice (Figure 1D). Since mechanical weakness of the BM is considered to be the cause of JEB, *Lamc2^jeb/jeb^* mice of each strain were subjected to a novel mechanical test that directly measures the force required to shear the tail skin epidermis from the dermis [21]. The shear force values correlated generally in an inverse manner with the overt skin lesion strain patterns and revealed phenotypic differences well before evidence of externally observable lesions (Figures 1E and S1B). Overall these results were consistent with the action of genetic background modifiers that act epistatically with the *Lamc2^jeb^* allele with varying strengths to alter the structural integrity of the skin, leading to substantial strain variation in the severity of clinical features of JEB.

### Identification of a major QTL on chromosome 19

**Figure 2:**
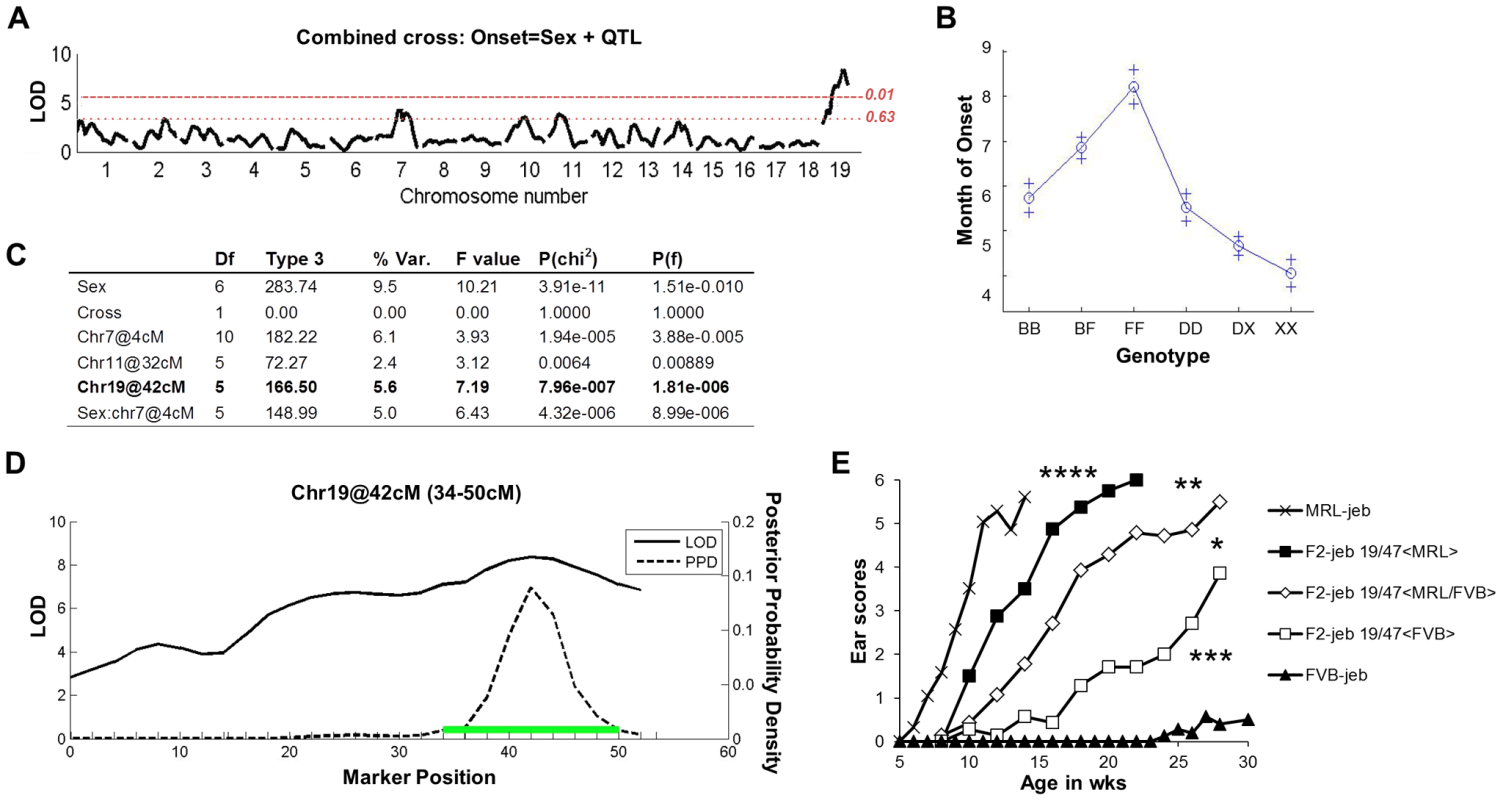
F_2_ segregation analysis identifies a strong chr19 QTL. **A**, Genome wide scan of a combined (DBA × 129X1) F_2_ *Lamc2^jeb/jeb^* (n = 243) and (B6 × FVB) F_2_ *Lamc2^jeb/jeb^* (n = 214) cross reveals a strong QTL on distal chr19. Horizontal dashed lines indicate suggestive (*P* = 0.63) and highly significant (*P* = 0.01) levels as determined by permutation testing. LOD, logarithm base 10 of odds. **B**, Effect plot for chr19 QTL. The y-axis indicates age of onset in months with standard error (+). X-axis is genotype (B, B6; F, FVB; D, DBA/1; X, 129X1). **C**, Multiple regression analysis. **D**, Expanded view of Chr19 QTL from combined cross. Green bar indicates the 95% confidence inteval. (2A–D) results from mixed sexes. **E**, Ear score severity of male (MRL × FVB) F_2_ *Lamc2^jeb/jeb^* mice correlates with chr19, 47 Mb SSLP marker *D19Dcr40*. Similarly significant genotype/phenotype correlations were found for SSLP markers at chr19, 32 Mb and chr19, 37 Mb and for female MRL × FVB F_2_ *Lamc2^jeb/jeb^* mice (data not shown). N = 7–29 F_2_ mice per genotype.

Crosses were made between phenotypically disparate strains to map the modifiers. Two groups of F_2_ *Lamc2^ jeb/jeb^* homozygotes were produced: (DBA1 × 129X1)-*Lamc2^ jeb/jeb^* F_2_ and (B6 × FVB)-*Lamc2^jeb/jeb^* F_2_, with >200 mice per group. Both sets of mice were monitored for the age at which ear lesions were first visually detectable and genome wide scans of their DNAs were performed using single nucleotide polymorphism (SNP) markers distributed through the autosomal and X chromosomes. For analysis, the F_2_ datasets were combined to increase the statistical power [22], and genome wide associations were determined using the R/QTL algorithm [23], [24]. Figure 2A–D presents the combined QTL analyses. Given that males develop disease before females, there was a strong sexual dimorphism (Prob>chi^2^ 3.9×10^−11^). Three QTLs were significant in multiple regression tests: chr19@42cM; chr11@32cM; and sex by QTL interaction (Sex*chr7@4cM). The sex*chr7@4cM QTL was observed only among the B6/FVB heterozygous mice. The chr11@32cM QTL dropped below the significance threshold if the sex*chr7@4cM QTL was excluded from the model, indicating it being a marginal QTL candidate. The most robust QTL (Prob>chi^2^ 7.96×10^−7^), replicated in both mouse crosses independent of sex and explaining 5.6% of the genetic variance, was located on chr19 with a 95% confidence interval of 34–50 cM peaking at 42 cM.

To determine if the chr19 QTL is replicated in the strain backgrounds showing the largest phenotypic differential (MRL and FVB), we performed genetic association studies on cohorts of (MRL × FVB) F_2_ *Lamc2^jeb^* homozygotes. Grouping the mice by the *D19Dcr40* SSLP marker mapping to ∼47 Mb on chr19 revealed a strong but incomplete concordance between *D19Dcr40* genotype and disease onset (males shown in Figure 2E; similarly significant patterns were observed for females: data not shown). Genotype/phenotype correlations for other chr19 SSLP markers were similar, failing to narrow the candidate interval (data not shown). These results provided further support for a complex genetic model, but with a chr19 QTL most robust and consistently replicated among different mouse crosses.

### Variation limited to chr19 impacts a spectrum of JEB phenotypes

**Figure 3:**
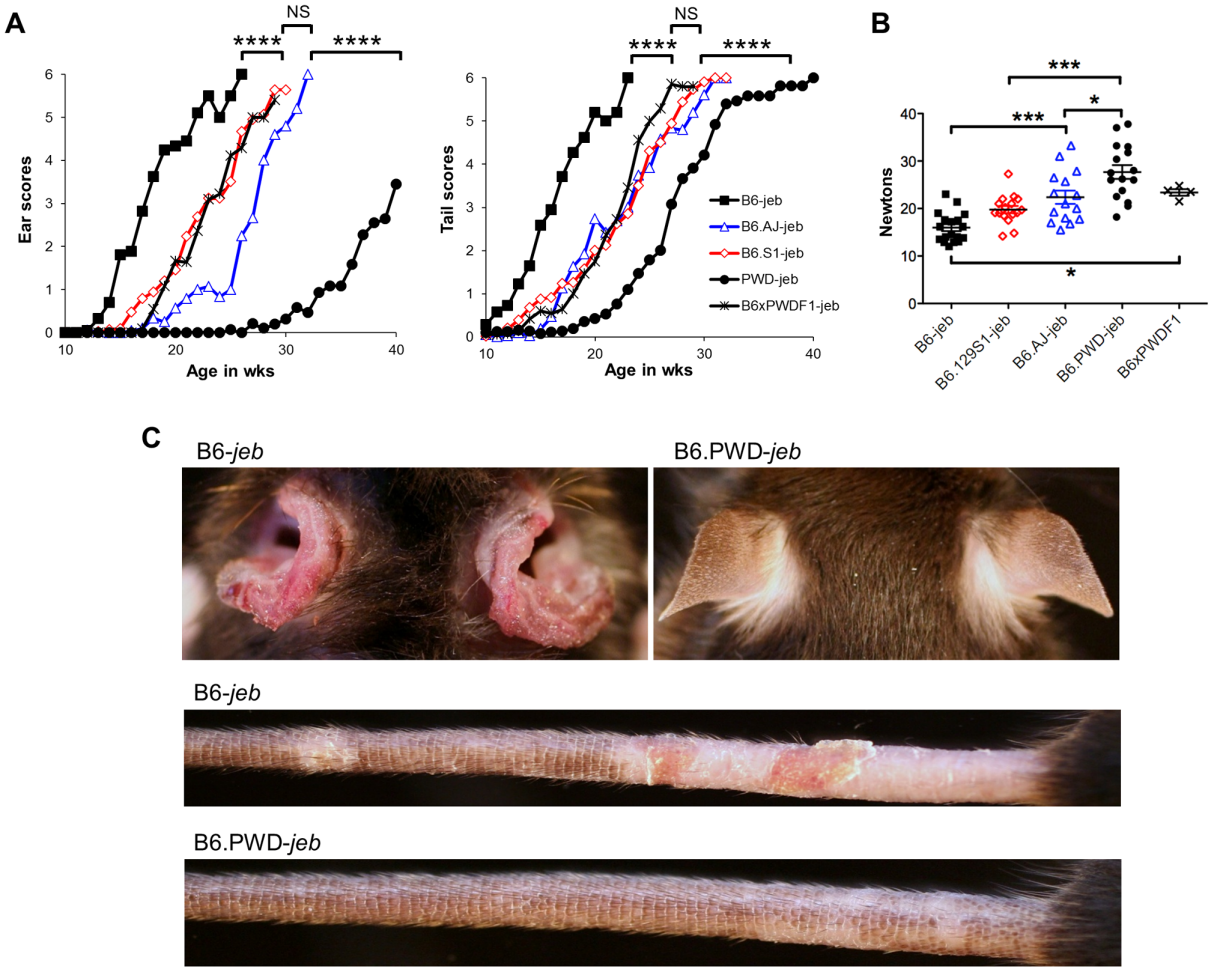
Allelic variation limited to chr19 influences the timing of onset of cutaneous phenotypes of JEB. **A**, Average ear and tail scores, and **B**, 10 week old tail tension test values comparing B6 mice (most susceptible) with B6.*chr19^PWD^* consomic (most resistant), B6.*chr19^A/J^* consomic, B6.*chr19^129S1^* congenic, and B6 × B6.*chr19^B6/PWD^* heterozygous male mice (intermediate resistance). **A**, N = 2 to 50 mice per timepoint. *, *P*≤0.05; ***; ≤0.001; ****, ≤0.0001; NS, >0.05. **C**, Representative images of ears and tails from 23 week old male B6 and B6.*chr19^PWD^* consomic mice. All mice are *Lamc2^jeb/jeb^*.

To more directly address the contributions of the chr19 QTL, experiments were designed in which genetic variation was limited solely to that chromosome. To do so, the *Lamc2^jeb^* allele was transferred onto existing chr19 consomic stocks B6.*chr19^PWD^* and B6.*chr19^A/J^* and a B6.*chr19^129S1^* congenic strain. Ear and tail scores and tail tension test comparisons indicated that the *chr19^A/J^* and *chr19^129S1^* alleles moderately attenuated disease and the *chr19^PWD^* allele strongly attenuated disease as compared to the *chr19^B6^* allele (Figure 3A–C). *Lamc2^jeb/jeb^* mice which were B6.*chr19^B6/PWD^* heterozygous were phenotypically midway between B6.*chr19^B6/B6^* and B6.*chr19^PWD/PWD^ Lamc2^jeb/jeb^* mice, demonstrating that the chr19 alleles interact in a co-dominant manner. Overall, the results demonstrated that allelic variation limited to chr19 was sufficient to discriminate three functional chr19 haplotypes with B6 being most susceptible, 129S1 and A/J being intermediate, and PWD being most resistant.

**Figure 4:**
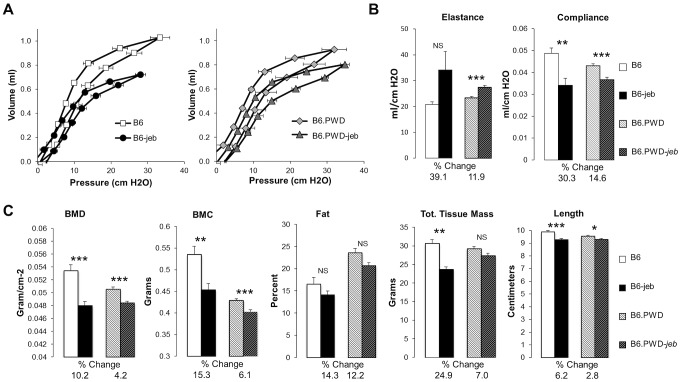
The PWD chr19 reduces respiratory and bone abnormalities of *Lamc2^jeb/jeb^* mice. **AB**, Lung pressure-volume (PV) loop comparisons (A) and lung resistance and compliance measurements (B) from 7–9 male B6 *wt*, B6-*Lamc2^jeb/jeb^*, B6.*chr19^PWD^ wt* and B6.chr19^PWD^ *Lamc2^jeb/jeb^* mice at 18–19 wks of age. **C**, Total bone mineral density (BMD), total bone mineral content (BMC), percent fat, total tissue mass, and body length from the same mice. *, *P*≤0.05; **, ≤0.01; ***, ≤0.001; ****, ≤0.0001 comparing *wt* and *Lamc2^jeb/jeb^* mice matched for chr19. Percent changes comparing *wt* and *Lamc2^jeb/jeb^* mice are indicated.

Non-cutaneous features of JEB also found in *Lamc2^jeb/jeb^* mice include compromised pulmonary function and low bone mineralization [20]. We addressed the impact of chr19 allelic variation on these non-cutaneous JEB phenotypes in the most phenotypically polarized chr19 strain comparison, B6 vs B6.*chr19^PWD^*. Forced oscillation pulmonary measurements showed that the B6.*chr19^PWD^* background attenuated pulmonary deficits, including reduced elastance and increased compliance, caused by homozygosity of *Lamc2^jeb^* as compared with the B6 background (Figure 4A–B). Dual-energy X-ray absorptiometry measurements indicated that the PWD chr19 ameliorated the reductions in bone mineral density, bone mineral composition, and non-bone tissue mass caused by homozygosity of *Lamc2^jeb^* while not affecting percentage of body fat (Figure 4C). Taken together, the results provide compelling support for chr19 modifier(s) that act epistatically with the *Lamc2^jeb^* mutation to control the adhesive strength of the BM and a spectrum of cutaneous and non-cutaneous phenotypes consistent with a JEB syndrome.

### The ‘protective genetics’ of B6.*chr19^PWD^* and FVB are insufficient to significantly impact the perinatal-lethal form of JEB caused by a null allele of *Lamc2*

Mice homozygous for the null *Lamc2^tm1Uit^* allele develop lethal JEB-H and do not survive beyond 5 days of birth [25]. Since FVB and B6.*chr19^PWD^* genetic backgrounds strongly ameliorated the disease of *Lamc2^jeb^* ^/*jeb*^ mice, we asked whether these backgrounds would prolong the lives of *Lamc2^tm1Uit/tm1Uit^* mice. The *Lamc2^tm1Uit^* allele was crossed onto the FVB and B6.*chr19^PWD^* backgrounds, and *Lamc2^tm1Uit/wt^* intercross matings were produced. Mendelian segregation predicts that ¼ of the pups would be homozygous for *Lamc2^tm1Uit^*. No *Lamc2^tm1Uit^* homozygotes survived to weaning. Thus, while strain backgrounds were able to modify the disease caused by the hypomorphic *Lamc2^jeb^* allele, they were insufficient to overcome the disease caused by a full genetic deficiency of *Lamc2* (Table 1).

**Table 1:** The FVB and B6.*chr19^PWD^* backgrounds do not rescue perinatal lethality of *Lamc2*-null mice.

Strain	*Lamc2* genotype*		
	+/+	*tm1Uit*/+	*tm1Uit*/*tm1Uit*
B6	51 (34%)	99 (66%)	0
B6.*chr19^PWD^*	49 (39%)	76 (61%)	0
FVB	36 (31%)	80 (69%)	0

* Genotyping results from progeny of *Lamc2^tm1Uit/+^* intercross matings of the indicated strain backgrounds.

+ . Wild-type.

### Recombinants map the Chr19 QTL to a 1085 bp fragment of *Col17a1*

**Figure 5:**
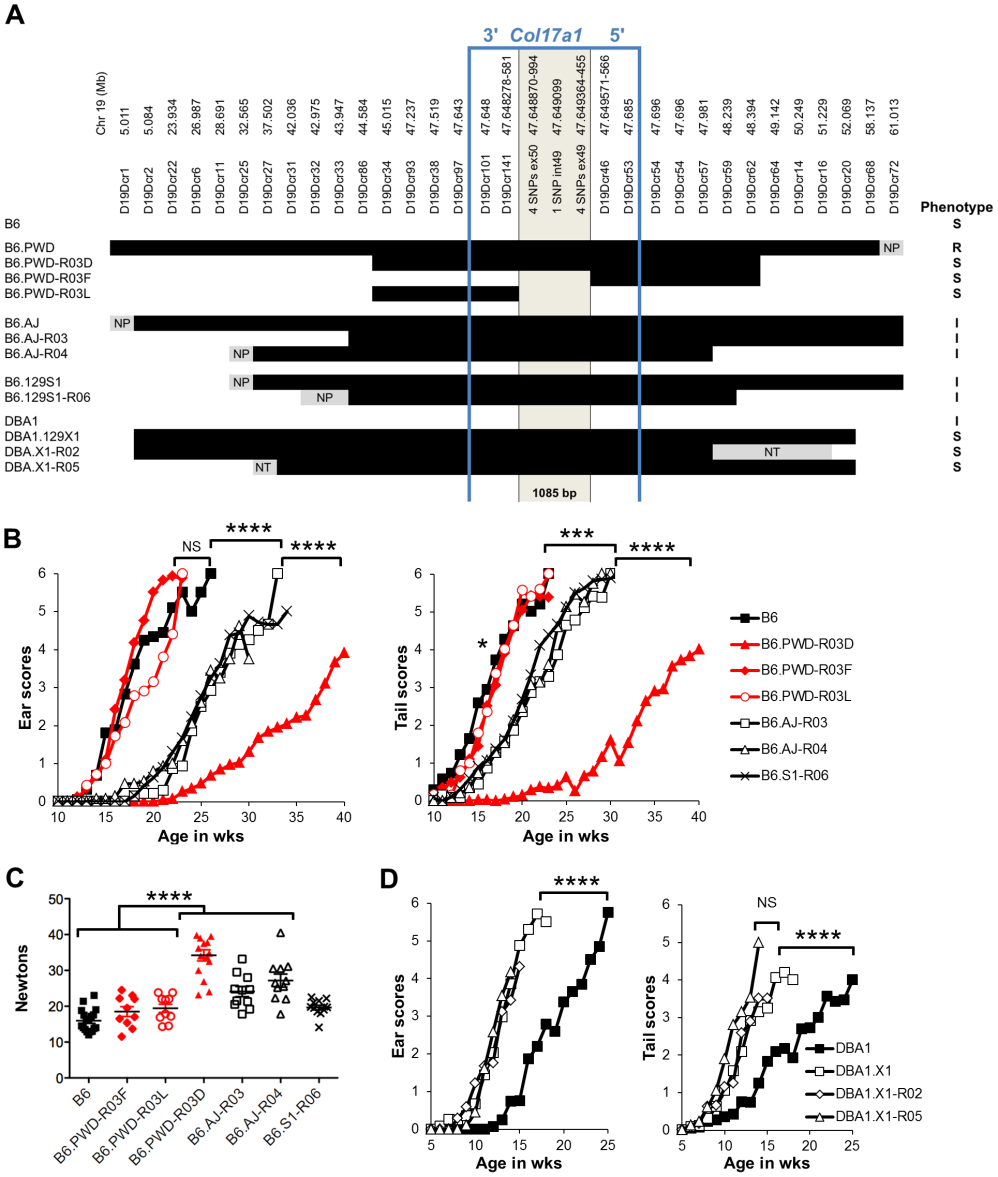
Reduced congenic mapping limits the modifier to within *Col17a1*. **A**, The most informative reduced congenics used in this study. Chr19 donor segments are indicated in black and background strain segments in white. Primer names and chr19 Mb positions are listed above based on C57BL/6J NCBI Build 38. NP, non-polymorphic; NT, not tested. Not to scale. Phenotypes: R, resistant; I, intermediate; S, susceptible. **BC**, Average ear and tail scores (B) and tail tension measurements at 10 weeks of age (C) for the most informative B6.*chr19^AJ^*, B6.*chr19^129S1^* and B6.*chr19^PWD^* recombinants. **D**, Ear and tail scores of DBA1.*chr19^129X1^* recombinants. ***, *P*≤0.001; ****, ≤0.0001, NS, >0.05.

To finely map the chr19 QTL, we used consomic and congenic reduction approaches. B6.*chr19^PWD^ Lamc2^jeb/jeb^* and B6-*Lamc2^jeb/jeb^* mice were crossed and a series of mice carrying reduced chr19 congenic segments and potentially informative breakpoints were identified from their progeny. Ear and tail score and tail tension tests were performed on cohorts of male mice homozygous for various recombinant chr19 haplotypes and *Lamc2^jeb/jeb^*. Sixteen recombinant strains with breakpoints along chr19 were analyzed phenotypically. In all cases they were consistent with a region including *Col17a1* as the chr19 QTL (Figure 5A–C and data not shown). This gene was an excellent candidate because mutations in human *COL17A1* can result in JEB [13], [26] and it lies within the major chr19 QTL intervals initially identified by the F_2_ mapping crosses. The B6.*chr19^PWD^* reduced congenic R03D showed a late onset PWD phenotype and thereby narrowed the candidate interval to <4.6 Mb including *Col17a1*. Two fortuitous intra-*Col17a1* recombinants, R03F and R03L, retained the PWD genotype for most of the candidate interval but each demonstrated the B6 early onset phenotype. The breakpoints of R03F and R03L, confirmed by SSLP and DNA sequencing, limited the interval to 1085 bp that included all of exon 49, intron 49 and exon 50 (Figure S2).

We also determined if the positioning of chr19 intermediate resistance alleles of strains A/J, 129S1, and DBA/1 were consistent with variation at the *Col17a1* locus. While not achieving the genetic resolution of the B6.*chr19^PWD^* consomic crosses, mapping of recombinant B6.A/J, B6.129S1 and DBA1.129X1 chr19 congenics limited the candidate intervals to <4.3 Mb, <6.4 Mb, and <19.6 Mb respectively, with each interval including all of *Col17a1* (Figure 5A–D). DBA1.*chr19^129X1^* results also confirmed the findings of the (DBA1 × 129X1) F_2_ mapping cross in which a chr19 QTL exists but the pattern is inverse to the overall strain phenotype: 129X1 is later onset than DBA1, but the *chr19^129X1^* allele confers earlier onset (Figure 2B; 5A, D). This indicates that other genetic modifiers must be at work to account for the overall DBA1 ‘earlier onset’ and 129X1 ‘later onset’ phenotypes, though no individual modifier was detectable by our QTL test. In all genetic contexts tested, the results were consistent with *Col17a1* being the chr19 QTL, with the B6 and 129X1 alleles being most susceptible, A/J, DBA1, and 129S1 being intermediate, and PWD being most resistant.

### The chr19 QTL is explained by 1–3 missense variants in exon 50 of *Col17a1*

To test whether modifier effects might be caused by variation in the expression of *Col17a1*, tail skin-derived cDNA from chr19 *Lamc2^jeb/jeb^* strains was tested by RT-qPCR at 6–8 weeks of age using oligonucleotide primer pairs designed to tile across the *Col17a1* transcript. We failed to detect expression differences for any of the *Col17a1* amplicons among the strains tested (Table S1). Therefore, differential expression of *Col17a1* or its isoform usage is unlikely to underlie the modifier effects of *Col17a1*.

To broadly evaluate the candidacy of sequence polymorphisms in *Col17a1* as modifiers, we sought to obtain complete sequence information for this gene for the eight relevant strains. We first queried the Mouse Phenome SNP Database (MPD, phenome.jax.org) to evaluate haplotype relationships in the region surrounding *Col17a1* (Table S2). These results justified the incorporation of B6, DBA/2 and PWK from Mouse Genomes Project queries (sanger.ac.uk) as representative of 129X1, DBA/1 and PWD respectively. Since FVB and MRL were not available in Sanger and were phenotypically extreme strains, we sequenced almost their entire *Col17a1* genes. cDNAs from 7 of the 8 strains (except 129S1) were also sequenced from exon 1 through most of exon 53. A comprehensive list of polymorphisms among the 8 strains is provided as Table S3.

**Figure 6:**
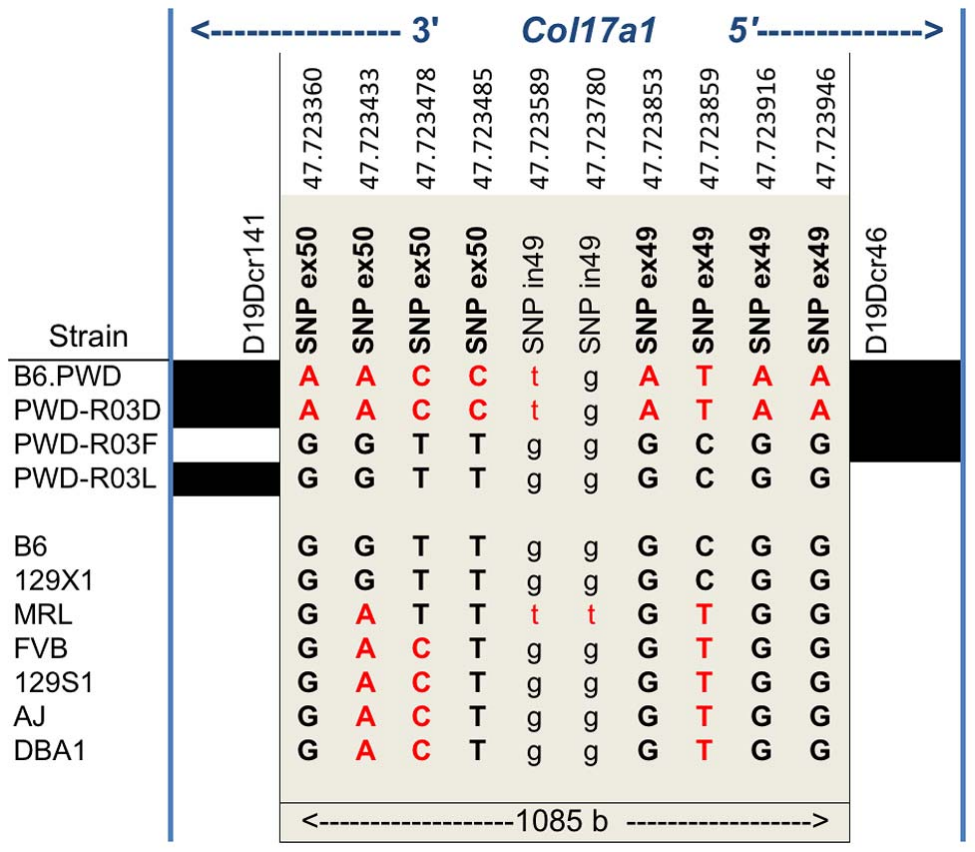
Nucleotide variation within the 1085*Col17a1* among phenotyped mouse strains. Positions of SNPs based on C57BL/6J NCBI Build 38 are indicated. Black, B6 SNP allele; red, other SNP allele;. **Bold**, uppercase exonic SNPs. Left and right blocks indicate the recombination boundaries of the candidate interval defined by B6/PWD breakpoints: white, B6; black, PWD.

None of the strains showed variation in *Col17a1* predicted to result in nonsense changes or altered splicing patterns. With the exception of PWD, there were no missense changes outside of the candidate 1085 bp interval of *Col17a1*. Limiting the analysis to the 1085 bp candidate interval defined by the PWD/B6 recombinants identified 3 non-synonymous coding SNPs (all in exon 50), 5 synonymous coding SNPs (4 in exon 49 and 1 in exon 50) and 1 intronic SNP that distinguished B6 from PWD (Figure 6). Aside from the three non-synonymous coding changes for PWD in exon 50, four missense SNPs were identified in other exons (Table S3). They were unique to PWD and ruled out as modifier candidates by the B6.*chr19^PWD^* recombinant strains R03F and R03L (Fig. 5A–D). The only missense SNPs identified in the remaining strains were allelic to the B6/PWD missense SNPs in the 1085 bp interval. Overall the results were consistent with the modifier effects being the result of coding polymorphisms within the candidate interval.

Translation of the three candidate missense SNPs identify amino acid (AA) positions 1275, 1277, and 1292 within the non-collagenous 4 (NC4) subdomain of collagen XVII. Four related haplotypes are evident: Haplo 1 (S^1275^ N^1277^ T^1292^) shared by B6 and 129X1; Haplo 2 (S^1275^ N^1277^ I^1292^) unique to MRL; Haplo 3 (S^1275^ S^1277^ I^1292^) shared by A/J, 129S1, DBA1 and FVB; and Haplo 4 (G^1275^ S^1277^ I^1292^) unique to PWD (Table 2). Comparisons of the haplotypes with overt skin lesion and dermal-epidermal adhesion phenotyping are most consistent with the key involvement of changes at positions 1275 and 1277, with N1277S resulting in intermediate resistance and S1275G+N1277S resulting in strong resistance. Taken together, the results support a concise explanation in which relatively conservative combinational variations limited to AA positions 1275 and 1277 are the chr19 modifiers.

**Table 2:** AA variation in the NC4 domain of mouse collagen XVII.

Haplotype	Strain	AA Seq (1275–1292)	Phenotype*
1	B6	**S** s **N** ssarrgtsyssstg **T**	S*
	129X1		
2	MRL	**S** s **N** ssarrgtsyssstg **I**	S#
3	129S1	**S** s **S** ssarrgtsyssstg **I**	I*
	A/J		I*
	DBA		I*
	FVB		I#
4	PWD	**G** s **S** ssarrgtsyssstg **I**	R*

S, most susceptible; I, Intermediate; R, most resistant.

* Based on phenotyping data of chr19 congenic *Lamc2^jeb/jeb^* mice.

# Inferred from analysis of F_2_ phenotyping (Figure 2E).

### Evaluation of the potential for *COL17A1* modifiers in humans

**Figure 7:**
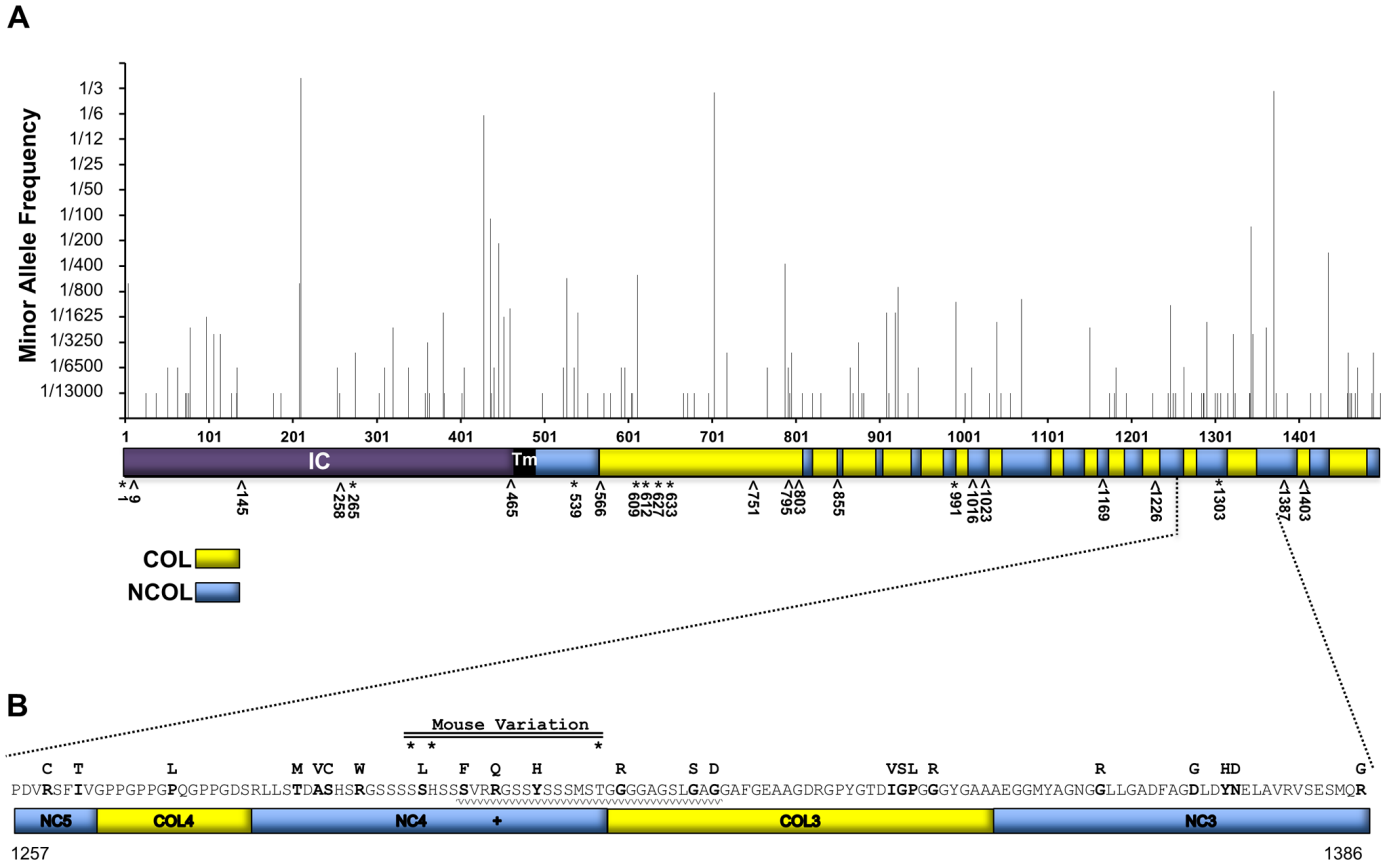
AA variation of human collagen XVII in a non-EB diagnosed human population in comparison with known JEB causing variants. **A**, Whole protein view of minority consensus AA allelic variant frequencies based on Exome Variant Server database and mapped to domains of human collagen XVII [30]. *, missense; ∧, nonsense JEB-causing mutations from HGMD. Blue, NC Domains; Yellow, COL domains based on *Giudice et al.* [30]. **B**, Predicted AA variation encoded by exon 52 of human *COL17A1*. AA sequence with minority allelic variants from EVS and 1000 Genomes indicated above in **bold**. **+**, known JEB causing missense variant, R1303Q [13], [41], [42]. *, positions of variants (mouse 1275, 1277 & 1292) aligned to human collagen XVII. Waved line indicates the region affected by the 4003delTC/4080insGG revertant mosaic double mutation causing the partial replacement of NC4 and partial loss of the COL3 domains [44].

Having identified certain coding variants of mouse *Col17a1* that modify JEB-nH in *Lamc2^jeb/jeb^* mice, we interrogated the Exome Variant Server (EVS, evs.gs.washington.edu) and the 1000 Genomes Project (1000GP, browser.1000genomes.org) human databases to assess the potential for coding variants in *COL17A1 -* in populations that were unbiased in that that did not including cases of documented EB - that may be candidates for imparting EB modifier effects. From the combined EVS+1000GP databases, representing ∼7600 individuals not known to be affected by EB, missense polymorphisms were identified for 13% (195 of 1497) of the codons of *COL17A1*. The minority allele frequencies of 135 missense polymorphisms among ∼6500 individuals (those reported in EVS) are shown in Figure 7A. The vast majority of these variants are rare, occurring in <1% of the population, but the four most common minority alleles represent 15, 28, 29 and 42% of the total allele counts.

To determine whether there are patterns to the allelic variation, the missense polymorphisms were analyzed for distribution across exons, protein subdomains, and the AA altered (Table S4). Seven of 56 exons contained no missense variants. Only exons 7 and 33 exceeded the average number of polymorphisms per length by >2 SD (Table S4A). They encode portions of the intercellular domain (IC) and collagenous (COL) segment 15, respectively. Of the protein segments (COL, NC, TM or IC), only NC5 differed in numbers of polymorphisms per length by ≥2 SD (Table S4B). Polyphen predicted that of the 135 missense *COL17A1* mutations reported in EVS, 15% were ‘possibly damaging’ and 47% ‘probably damaging’. However, such predictions can be misleading. The most common minority allele, leading to the change T210M, found in 42% of the population and homozygous in 20% of the individuals surveyed, is predicted by Polyphen to be ‘probably damaging’, but 20% of the population does not have EB. Substitutions for Glycine, Proline and Arginine were most frequent overall (Table S4C). Missense mutations in Glycine or Proline in the X position of G-X-Y triplets are the AA changes considered to be most likely to alter collagen XVII function and cause JEB [13], [27], [28]. Such substitutions did not exceed frequency expectations, as the collagenous structure contains many G-X-Y repeats, particularly G-P-H [29], [30]. Only Arginine was substituted at a frequency higher than would be expected by chance.

Figure 7A also indicates the locations of known missense (9) and nonsense (15) JEB causing mutations in *COL17A1* from cited publications and as summarized in the Human Gene Mutation Database (HGMD, hgmd.cf.ac.uk). No JEB-associated nonsense mutations were found in the EVS or 1000GP databases, perhaps due to their deleterious effects. However, three known JEB-causing missense transpositions (G633D, V991M and R1303Q) segregating in the general population at low frequency in a heterozygous state. EVS and 1000GP also record missense variants in 6 of 15 AA positions that overlap with JEB-causing nonsense mutations (positions 9, 258, 566, 795, 803 and 1226); the consequences of this overlap are not known.

Figure 7B focuses on the translated product of human exon 52, which is the equivalent of mouse exon 50 and the interval to which we document modifier affects in *Lamc2^jeb/jeb^* mice. A number of JEB causing mutations have been reported to locate to exons 51 and 52 [13]. Human exon 52 encodes part of NC5, all of COL4, NC4 and COL3, and part of the NC3 domain [30]. Notably, NC4 carries the JEB causing R1303Q coding mutation, which aligns in close proximity to the 3 putative mouse JEB modifier variants we have identified. Numerous additional missense variants resulting in non-conservative AA changes are found in this interval. Extrapolating from our findings in mice, such missense variants may be candidates for genetic modifiers of JEB and potentially other forms of EB.

## Discussion

We have addressed the prospect of genetic modifiers of a heritable, Mendelian mechanobullous disorder. Using mice homozygous for the *Lamc2^jeb^* mutation and varying normally asymptomatic background genetics, we document the potent impact of genetic modifiers on a form of JEB-nH. Through an unbiased genetic approach involving a combination of QTL mapping and positional cloning, we unambiguously show that the gene *Col17a1* is a strong genetic modifier of the non-Herlitz form of JEB that develops in *Lamc2^jeb^* mice. This modifier is explained concisely by variations in 1 to 3 neighboring AA in the NC4 domain of the collagen XVII protein that are normally innocuous but act epistatically to alter the strength of dermal-epidermal adhesion in the context of the *Lamc2^jeb^* mutation and broadly impact the clinical severity of JEB.

### Genetic modifiers can substantially impact the expression of JEB caused by the *Lamc2^jeb^* mutation

The pathophysiology of the spontaneously arising *Lamc2^jeb^* mutation was originally described in the context of the129X1/SvJ genetic background on which the mutation was first detected [20]. Consistent with JEB-nH, this included progressive skin blistering caused by separations at the lamina lucida of the basement membrane. While core manifestations of JEB were evident in *Lamc2^jeb^* homozygotes in the context of multiple genetic backgrounds, a remarkable range of variation in their age of onset and overall severity was apparent. In the most extreme cases, the MRL background presented with maximal, life threatening disease scores by 10 weeks of age, while FVB did not achieve maximum scores until >47 weeks of age. Other strains demonstrated intermediate phenotypes, all supporting a typical, complex genetic model in which several allelically variant loci contribute to overall disease.

However, a unifying feature was their modes of action. Genetic variables envisioned to impact JEB include: 1) those that integrally contribute to the structural elements of the basement membrane; and 2) those that act secondarily through inflammation or repairing skin lesions (including loci that affect wound healing). The fact that measurements of the strength of dermal-epidermal adhesion was predictive of disease onset and severity well before the appearance of dermal lesions and that the excellent wound healing strain background, MRL [31], was most susceptible to JEB strongly indicates the former model in which the genetic modifiers act intrinsically to progressively affect the structural integrity of the dermal/epidermal layer. If this principle can be extrapolated to humans, measurement of the strength of dermal/epidermal adhesion at an early age may have prognostic value. It is noted, however, that genetic background modifiers did not detectably impact the perinatal lethal, Herlitz form of JEB caused by the complete loss of function *Lamc2^Uit^* allele. This dichotomy likely reflects a common feature of ‘monogenic’ disorders: mutations that result in profoundly life threatening consequences will overshadow potentially modulating factors, whereas mutations in disease causing genes that result in more attenuated forms, such as the JEB-nH syndrome of *Lamc2^jeb^* mice, are more susceptible to the effects of background modifiers.

### Allelic variants in the NC4 domain of collagen XVII are strong modifiers of JEB in mice

The results described herein identify relatively conservative AA changes limited to the NC4 domain of collagen XVII that are able to modulate a JEB syndrome caused by a hypomorphic mutation in the LAMC2 component of the laminin 332 complex. Multiple lines of evidence triangulated to *Col17a1*: 1) mapping in two F_2_ crosses (DBA1 × 129X1-*Lamc2^ jeb/jeb^* F_2_ and B6 × FVB-*Lamc2^jeb/jeb^* F_2_) identified a strong QTL overlapping the *Col17a1* locus; 2) phenotype to *Col17a1* genotype correlations of F_2_ mice of the most polarized strain backgrounds (MRL and FVB) were highly significant; 3) phenotypic analysis of chr19 congenics and sub-congenics consistently framed *Col17a1* as the modifier locus. Limitation of the disease modifying effects to neighboring coding changes within exon 50 of *Col17a1* was strongly indicated by fortuitous recombinants from crosses of B6 and B6.*chr19^PWD^* mice that limited the chr19 candidate interval to 1085 bp and included three non-synonymous coding SNPs.

This deep genetic resolution is unusual. PRDM9 is a long-array zinc-finger and chromatin-modifier protein that binds DNA sequences and promotes their meiotic recombination [32], [33]. Indeed, evaluation of *Col17a1* using the Persikov – Singh algorithm [32] identifies a PRDM9 binding motif in the center of the recombinant interval (Table S5). It is therefore likely that the unusual genetic resolution achieved was facilitated by a recombination hotspot.

The candidacy of these missense SNPs as the cause of the JEB-modifying chr19 QTL among a number of mouse strains analyzed is strongly supported by sequence comparisons. The missense SNPs predict variation in neighboring AA positions 1275, 1277, and 1292 in the NC4 domain (Table 2). Comparisons using overt skin lesion scoring data are most consistent with the key involvement of changes at two positions, 1275 and 1277, with N1277S resulting in the shift to intermediate resistance and S1275G+N1277S, unique to PWD, resulting in strong resistance. Strongly correlated patterns, as measured by changes in the strength of dermal-epidermal adhesion, indicate that these polymorphisms directly alter the adhesive strength of the dermal-epidermal junction in *Lamc2^jeb/jeb^* mice. Moreover, it is likely that the modulatory effects of these variations are not limited to the cutaneous layer because the PWD chr19 conferred more resistant to non-cutaneous manifestations of JEB, including pulmonary functions and bone mineralization. Thus, seemingly subtle AA changes in the collagen XVII NC4 domain modify multiple pathophysiological manifestations of a JEB syndrome caused by a primary defect in a component of laminin 332.

### How might AA variants limited to the NC4 domain of collagen XVII modify JEB caused by a mutation in a component of the laminin 332 complex?

Our results support a model of functional epistasis in which a mutationally crippled form of laminin 332 permits normally innocuous AA changes to become functionally significant modifiers of JEB. The components of laminin 332 form an extracellular matrix heterotrimer that tethers the epidermal and dermal layers. The heterotrimer is secreted by keratinocytes assembled as N-terminal branching from a central coiled-coil structure with globular domains at its C-terminus. The standard model is that this heterotrimer bridges the lamina lucida and lamina densa by binding of its C-terminal globular domains to membrane bound α6ß4 integrin of basal keratinocytes and its N-terminus β3 and γ2 arms to collagen VII anchoring fibrils in the dermis [11], [17], [34]–[36]. Thus, loss-of-function or hypomorphic mutations in any one of the laminin 332 components that affect the functions or abundance of the heterotrimer could theoretically reduce its connectivity to α6ß4 integrin, collagen VII or other binding partners and potentially cause JEB.

A product of basal keratinocytes, collagen XVII forms a homotrimeric type II transmembrane molecule whose extracellular portion is comprised of 15 COL domains that presumably provide rigidity separated by 16 NC domains [30], [37]. Typically private mutations ranging from insertions and deletions to nonsense and missense mutations over the entire length of the human *COL17A1* gene can by themselves result in clinical manifestations of JEB-nH with varying severity. The carboxyl-terminal half of the collagen XVII extracellular domain has been shown to interact with laminin 332 [38]–[40], which provides evidence for biochemical linkage. Direct JEB-causing alterations in the NC4 domain have also been described. The recessively acting missense mutation, R1303Q [13], [41], [42] and the 4003delTC/4080insGG mutation (which substitutes 25 erroneous AA into the NC4-COL3 domains) cause forms of JEB presumably by conformational alterations of the NC4 domain [43], [44] (Figure 7B). A disease-relevant genetic linkage between *COL17A1* and *LAMB3* has also been reported where the proband was a compound heterozygote for L855X and R1226X *COL17A1* nonsense mutations and heterozygous for the R635X recessive *LAMB3* nonsense mutation [45]. The results overall are consistent with the need for interactions between laminin 332 and collagen XVII to maintain strong basement membrane zone adhesions, with conformational properties of the collagen XVII NC4 domain being particularly critical for this interaction. The functional epistasis we report shows that subtle AA changes at key contacts in the NC4 domain modify JEB when the laminin 332 complex is mutationally crippled. However, we additionally raise the possibility that these *Col17a1* modifiers may not be limited in their effect to diseases caused by mutant forms of laminin 332. In this context, it is of interest that QTL genetic analysis of mice that are experimentally induced to develop a form of EB Acquisita after immunization with a collagen VII polypeptide identified a strong QTL whose position overlaps with *Col17a1* [46]. It is therefore possible that *Col17a1* modifiers of JEB may also be able to impact other forms of blistering diseases.

### Are genetic modifiers operative in human JEB?

The existence of remarkably potent genetic modifiers of JEB in mice raises the possibility that genetic modifiers may also contribute to the spectrum of clinical variation noted for many subforms of EB in humans. At the highest level, the considerable phenotypic and clinical variability of EB is explained by the fact that mutations in at least 18 genes can lead to these mechanoblistering disorders. The second level of variation is caused by the nature of each mutation and its penetrance. The third level, which is the focus of our study, is the extent to which missing heritability is caused by genetic modifiers. Potent modifiers of JEB are evident among strains of laboratory mice despite their simple genetic structure in which public allelic variation prevails because of related heritages and inbreeding. Humans, in contrast, have a much deeper haplotype structure with a spectrum of variation ranging from public to rare alleles and confounding genotype/phenotype associations. This is further complicated by the rarity of human cases for any given EB disease-causing mutation and lack of reliable and comparable phenotype information concerning those affected. Our survey of the *COL17A1* gene in the EVS and the 1000GP databases, unbiased by cases of documented EB, identifies numerous missense SNPs, including those that encode AA that map within and around the functionally-documented NC4 domain of collagen XVII. Such candidate modifier SNPs would be likely bypassed by conventional mutation detection-biased methods, but may act epistatically with the disease causing mutation as genetic modifiers. Thus, informed by mouse studies and focusing genetic modifier searches on allelic variants within functionally-important interaction domains of dermal-epidermal adhesion proteins may aid in the genetic prognosis and diagnosis of mechanobullous blistering disorders. Furthermore and as exemplified by this study, the elucidation of modifier loci may provide new clues into the functionally relevant interactions among these proteins that could direct new treatments for EB.

## Materials and Methods

### Ethics statement

All animal protocols were reviewed and approved by The Jackson Laboratory Institutional Animal Care and Use Committee (approval #01022).

### Mice

129X1-*Lamc2^jeb/jeb^* and B6-*Lamc2^jeb/jeb^* homozygous for the hypomorphic *Lamc2^jeb^* allele were described previously [20], [21]. DBA1-*Lamc2^jeb/jeb^*, FVB-*Lamc2^jeb/jeb^* and MRL-*Lamc2^jeb/jeb^* were produced after backcrossing the 129X1-*Lamc2^jeb^* allele for 10 generations onto DBA/1J, FVB/NJ and MRL/MpJ strain backgrounds and selecting to be homozygous for a congenic interval including the 129X1-defined *Lamc2^jeb^* allele framed by markers *D1Dcr2* and *D1Dcr15* located at 150 and 160 Mb on chr1. *Lamc2^jeb/jeb^* mice consomic for chromosome 19 were produced by crossing the *Lamc2^jeb^* allele from B6-*Lamc2^jeb/jeb^* mice to C57BL/6J-*chr19^A/J^*/NaJ and C57BL/6J-*chr19^PWD/Ph^*/ForeJ to produce strains B6.*chr19^A/J^ Lamc2^jeb/jeb^* and B6.*chr19^PWD^ Lamc2^jeb/jeb^*. The chr19 congenic strain B6.*chr19^129S1^ Lamc2^jeb/jeb^* was similarly produced by a cross of B6-*Lamc2^jeb/jeb^* to B6.129(Cg)-*Pnlip^tm1Dyh^*/J mice. The *Pnlip^tm1Dyh^* targeted mutation was originally made using (129X1/SvJ × 129S1/Sv)F_1_-*Kitl^+^*-derived R1 embryonic stem cells [47]. Genotyping at markers polymorphic between 129X1 and 129S1 indicated that the mutation integrated into 129S1-derived chromatin and that B6.*chr19^129S1^ Lamc2^jeb/jeb^* mice carry a large 129S1-derived chr19 congenic segment (Figure 5A). Chr19 congenic DBA1.*chr19^129X1^* mice homozygous for *Lamc2^jeb^* mice were produced after backcrossing the chr19 segment from 129X1 to DBA1-*Lamc2^jeb/jeb^* for 10 backcross generations. Informative chr19 recombinant congenic and subcongenic stocks were generated by genotyping potentially recombinant progeny for SSLP using standard procedures. For examination of mice lacking *Lamc2*, the targeted null *Lamc2^tm1Uit^* allele [25] was backcrossed for 10 generations to C57BL/6J to produce B6-*Lamc2^tm1Uit/+^* mice. B6-*Lamc2^tm1Uit/wt^* was crossed to C57BL/6J-*chr19^PWD/Ph^*/ForeJ to produce B6.*chr19^PWD^ Lamc2^tm1Uit/wt^*. FVB-*Lamc2^tm1Uit/wt^* mice were produced by backcrossing the *Lamc2^tm1Uit^* allele from B6 onto FVB/NJ mice for 9 generations. *Lamc2^tm1Uti/+^* intercross matings were made using each of these three strain backgrounds to evaluate the survivability of *Lamc2^tm1Uit/tm1Uit^* homozygotes. All mice were bred and maintained in a specific pathogen free low barrier animal room under standard husbandry conditions.

### Genotyping

All crosses which required identification of *Lamc2^jeb^* genotype were identified by SSLP typing of the bracketing markers *D1Dcr2* and *D1Dcr15* (informatics.jax.org) on chr1 at ∼150 and 160 Mb, respectively. Primers were also developed to identify both *Lamc2 wt* and *jeb* mutant bands to confirm homozygosity. *Lamc2Dcr1a* F- CCTGTCTCATTTCTGGTAGGCTTT and R-CACACGTCACCACACCTGCT oligonucleotide primers mapping to intron 18, and *Lamc2Dcr1b*- F-GCGCCAGTCCTCCGATAGA mapping to the retroviral insert were used in a three primer reaction, giving bands of 121 bp for wild-type and 218 bp for *Lamc2^jeb^*. Primers used to identify *Lamc2^tm1Uit^* wild-type and mutant bands were as previously described [25]. SSLP markers listed in Table S6 were used for genotyping of chr19 congenic mice. They were designed using Primer Express 1.0 or 2.0 software (Applied Biosystems, Inc.) to bracket regions of dinucleotide repeats or known B6/PWD indels identified in Ensembl C57BL/6J reference sequence, with annealing temperatures of 58–60°C and reference product sizes of ∼100 bp.

### Ear and tail phenotype scoring

Mice were typically scored weekly. Ears were scored from 0 (unaffected) to 6 (severe) based on limited or overall inflammation and ear erosion. Tails were scored from 0 (unaffected) to 6 (severe) based on the generalized loss of epithelial structure, scarring, and scabbing. Time to event survival analysis using Cox proportional hazards model was used to identify strain/genotype differences on ear and tail score across different cohorts of mice. Time to event was defined as age at which subjects first reached an ear/tail score of 4. When multiple levels of strains/genotype existed within a given cohort, risk ratios were calculated across all pairs of strains to determine significant differences among strains/genotypes at *P*<0.05.

### Tail tension test

Determination of the mechanical force in Newtons necessary to cause the dermal-epidermal cleavage of tail skin was performed on euthanized mice as previously described [21]. One-way ANOVA analyses were used to test for differences among the five strains of mice for tension scores. Residuals of each ANOVA analyses were inspected to confirm that the ANOVA modeling assumptions were met. If a significant effect of strain was observed, a Tukey's HSD post-hoc test was performed to determine significant differences among strains at *P*<0.05.

### QTL mapping

For QTL analysis, a combined cross of 243 (DBA/1J × 129X1) F_2_ *Lamc2^jeb/jeb^* and 213 (B6 × FVB/NJ) F_2_ *Lamc2^jeb/jeb^* mice were analyzed in this study. Age of onset in weeks was identified for each animal with censored values for those ‘unaffected’ by 12 months of age. Genetic cohorts were genotyped for 147 or 136 SNP markers across the autosomal and X chromosomes spaced approximately ∼13 Mb through the Jackson Laboratory Fine Mapping Service by KBiosciences (Hoddesdon, UK). Data from the two crosses were combined in Pseudomarker2.02 [24]. Pseudomarkers generated at 2 cM spacing for each chromosome for one dimensional genome scans were performed using 128 imputations [24]. One thousand permutations were performed to determine the thresholds for QTL detection [48] and genome wide associations were determined using the R/QTL algorithm [23], [24]. QTL with LOD scores above 1%, 5%, 10% and 63% thresholds were calculated from the permutation results [49]. Genome-wide two-dimensional scans were then performed for associations between all possible pairs of QTL locations and lesion scores as described. QTL and possible QTL*QTL interactions identified from a single QTL scan and pair wise scan were fit into multiple regression models. *P* values for terms in the multiple regression model were calculated, and terms were dropped sequentially until all of the terms in the model were significant at the 1% level for main QTL effects and 0.1% for interaction effects.

### Immunofluorescence imaging

Radial tail sections were cut with a razor blade, frozen in OCT on dry ice, and then stored at −40°C. Samples were sectioned between 10 and 12 µm thick onto super frost plus slides (Fisher Scientific, Pittsburgh, PA). The sectioned tissues were fixed in ice-cold acetone for 10 minutes, washed three times in phosphate-buffered saline, and then allowed to dry. Primary antibodies were added to slides pre-blocked with 3% horse serum for 2 hours at room temperature in a humid chamber. The slides were then washed 3× in phosphate-buffered saline, incubated in the dark with fluorescence labeled secondary antibodies for 1 hour, washed 3× and cover-slipped using anti-fade gel mounting media (Sigma-Aldrich, St Louis, MO). Rabbit anti-mouse collagen VII (a gift from Z. Liu, University of North Carolina) diluted 1∶50 and rat anti-mouse integrin α6 (GeneTex, San Antonio, TX) diluted 1∶100 were used for immunostaining. Secondary antibodies used at a dilution of 1∶250 were goat anti-rabbit FITC and goat anti-rat Alexa Fluor 546 (Invitrogen/Molecular Probes, Carlsbad, CA). Imaging was performed using a SP5 Leica confocal microscope at 63× (Leica Microsystems, Bannockburn, IL).

### FlexiVent pulmonary analyses

Mice were anesthetized with ketamine/dormitor at doses based on body weight. To prevent the mice from breathing against the respirator, they were treated with pancuronium bromide in NaCl as a muscle relaxant at 0.2 mg kg^−1^. Once the mouse was anesthetized, a small incision was made between the third and fifth tracheal rings and a tracheal cannula was inserted and secured in place with suture. Each mouse was ventilated at 200 breaths per minute with a tidal volume of 10 ml kg^−1^ body weight using a flexiVent ventilator (SCIREQ, Montreal, Canada). Once the mouse was breathing passively, two consecutive sigh breaths were performed to open the airways and lungs. Sigh breaths were performed throughout the experiment to insure that the airways remained open. Pressure-volume loops, elastance and compliance measurements were recorded and analyzed using SCIREQ flexiVent 5.1 software. Comparisons between cohorts were performed using the Student's two-tailed heteroscedastic t-test.

### Dual-energy X-ray absorptiometry

Peripheral dual-energy X-ray absorptiometry performed using a Lunar PIXImus densitometer (GELunar, Madison WI) was used to assess bone and non-bone tissue parameters. Bone parameters of mineral density (BMD) and mineral composition (BMC), and non-bone parameters of percent fat and total tissue mass (lean+fats) were determined after head exclusion and analyzed using Lunar PIXImus 2.1 software, as described [20]. Comparisons between cohorts were performed using the Student's two-tailed heteroscedastic t-test.

### Gene expression analysis by RT-qPCR

Skin of uniform size was removed from the ventral tail of euthanized 6–8 week old female mice, collected in RNAlater (Qiagen), stored at room temperature for 24 hours, and then frozen at −40°C until ready to process. Total RNA was isolated using the standard Trizol reagent method (Invitrogen). Total RNA (500 µg) was converted to cDNA using the MessageSensor RT kit (Ambion/Applied Biosystems). *Col17a1* specific oligonucleotide primers pairs for RT-qPCR (Table S1B) were designed using Primer Express software (Applied Biosystems, Inc.). Expression quantification was performed using standard procedures with an ABI Prism 7900HT (Applied Biosystems).

### DNA sequencing and analysis

Exonic DNA sequences were derived from cDNA transcribed from tail skin amplified by *Col17a1* specific oligonucleotide primers using a Qiagen LongAmp PCR kit according to the manufacturer's protocols. Genomic DNA obtained from blood processed similarly was used to obtain intron-spanning sequences. PCR products were magnetic bead purified and sequenced on an Applied Biosystems 3730xl by the JAX DNA Sequencing Service. Sequence data were analyzed using Applied Biosystems Sequencing Analysis software version 5.2 and Sequencher software version 4.10. The Sanger Mouse Genomes Project (sanger.ac.uk) was used as the source of complete genomic sequence comparisons of strains including C57BL/6J, A/J, 129S1/SvImJ, DBA/2J and PWK/PhJ. The Mouse Phenome Database (phenome.jax.org) CGD-MDA1 dataset was utilized to compare strain polymorphism patterns in *Col17a1* and its flanking regions. These data were combined with our sequence and SSLP typing data related to 129X1, DBA/1J and PWD to identify all *Col17a1* polymorphisms among 8 strains (Table S3).

## Supporting Information

Figure S1 Mouse strain background causes substantial variation in the onset and severity of JEB-nH in *Lamc2^jeb/jeb^* mice. **A**, Average ear and tail scores from female *Lamc2^jeb^* ^/j*eb*^ homozygotes demonstrate a range of time of onset. **B**, Average tail tension measurements (in Newtons) in mice 10 weeks of age for the same strains (datapoints are measurements for individual mice). *, *p*≤0.05; ***; ≤0.001; ****, ≤0.0001. Statistical significance of tail scoring data is not provided because most groups of mice were euthanized before achieving a score of 4 due to the severity of their ear lesions.
(TIF)

Figure S2 Gel validation of B6/B6.*chr19^PWD^* recombinants. * indicates the recombination breakpoints that frame the candidate interval.
(TIF)

Table S1 RT-qPCR fails to identify transcriptional alterations in tail skin *Col17a1* expression among mouse strains surveyed.
(DOCX)

Table S2 Strain relationships of the genomic region including *Col17a1* among relevant mouse strains.
(DOCX)

Table S3 Sequence-based polymorphisms of *Col17a1* among the mouse strains used in this study.
(XLSX)

Table S4 Amino acid polymorphisms of human Collagen XVII based on the EVS and 1000 genome databases.
(DOCX)

Table S5 Localization of a PRDM9 binding site in the 1085 bp recombinant interval of *Col17a1*.
(DOCX)

Table S6 Chr1 and Chr19 markers used in this study.
(XLSX)
