# Supplementary figures and images for: Molecular Identification of Collagen 17a1 as a Major Genetic Modifier of Laminin Gamma 2 Mutation-Induced Junctional Epidermolysis Bullosa in Mice
Source: PLoS Genet. 2014 Feb 13;10(2):e1004068. doi: 10.1371/journal.pgen.1004068 (PMC3923665; doi:10.1371/journal.pgen.1004068)

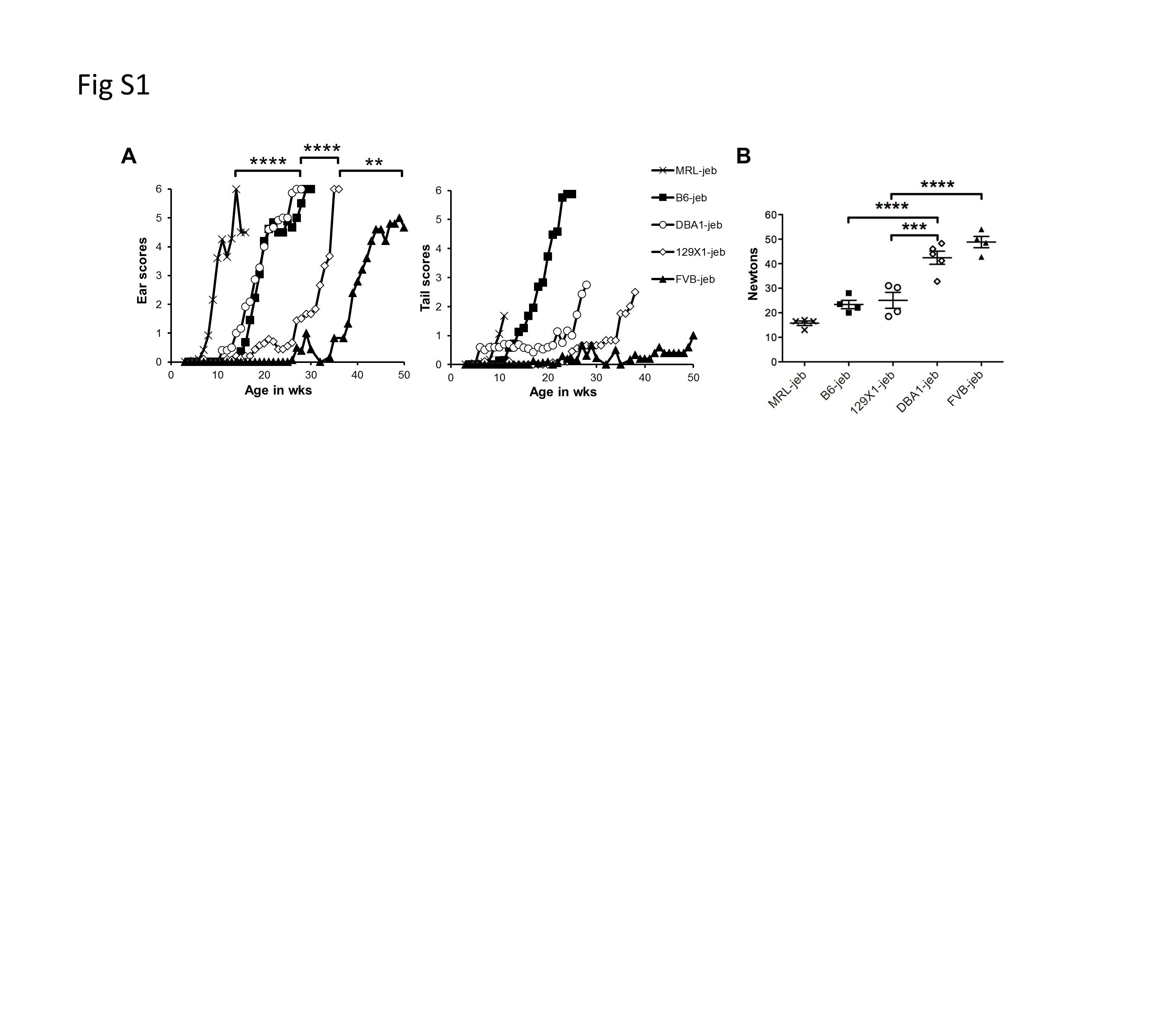

Supplement: Figure S1 — Mouse strain background causes substantial variation in the onset and severity of JEB-nH in Lamc2jeb/jeb mice. A, Average ear and tail scores from female Lamc2jeb /jeb homozygotes demonstrate a range of time of onset. B, Average tail tension measurements (in Newtons) in mice 10 weeks of age for the same strains (datapoints are measurements for individual mice). *, p≤0.05; ***; ≤0.001; ****, ≤0.0001. Statistical significance of tail scoring data is not provided because most groups of mice were euthanized before achieving a score of 4 due to the severity of their ear lesions. (TIF) [file pgen.1004068.s001.tif]

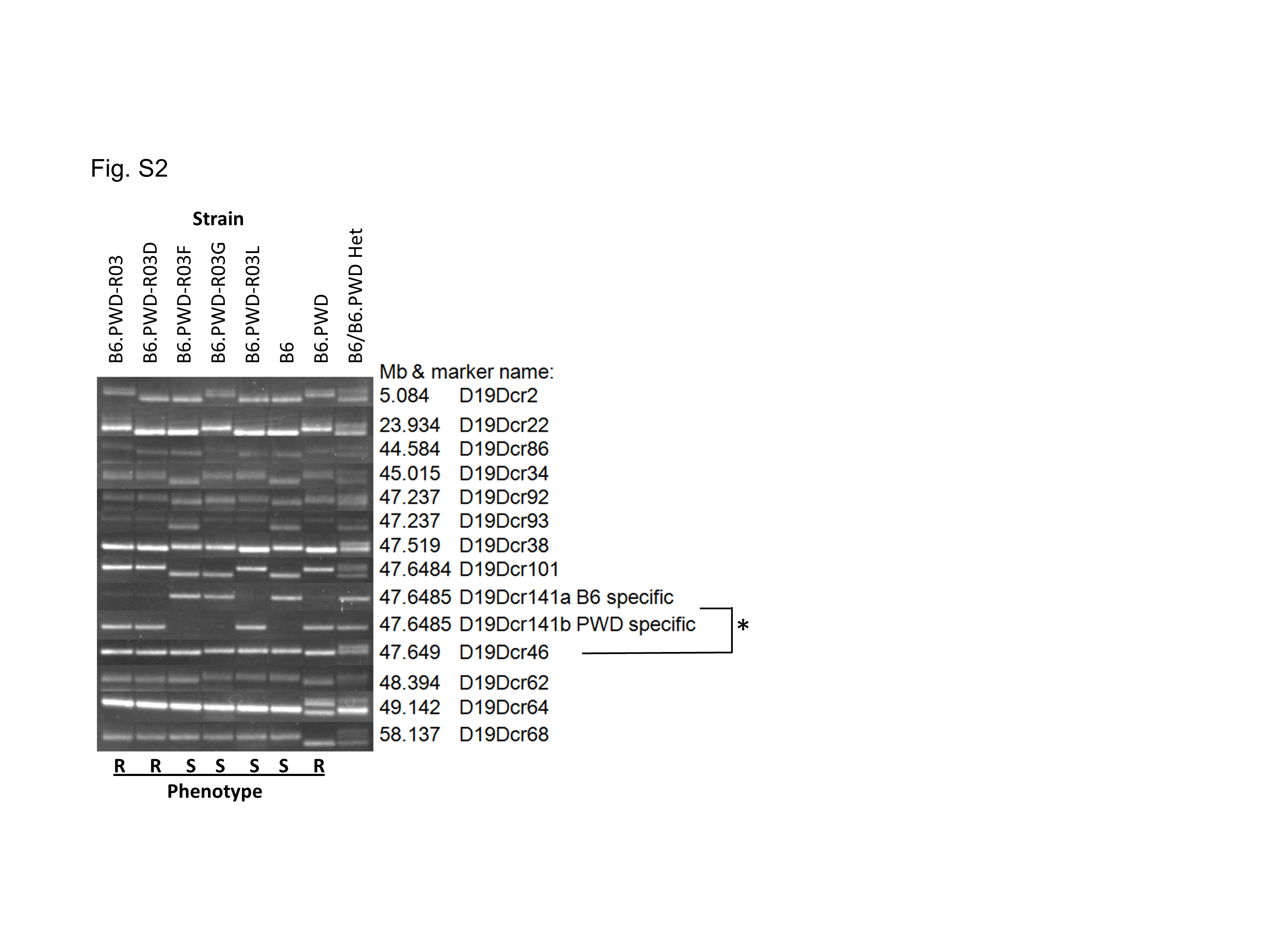

Supplement: Figure S2 — Gel validation of B6/B6.chr19PWD recombinants. * indicates the recombination breakpoints that frame the candidate interval. (TIF) [file pgen.1004068.s002.tif]
