# Supplementary material for: Molecular Identification of Collagen 17a1 as a Major Genetic Modifier of Laminin Gamma 2 Mutation-Induced Junctional Epidermolysis Bullosa in Mice
Source: PLoS Genet. 2014 Feb 13;10(2):e1004068. doi: 10.1371/journal.pgen.1004068 (PMC3923665; doi:10.1371/journal.pgen.1004068)
Supplement: Table S1 — RT-qPCR fails to identify transcriptional alterations in tail skin Col17a1 expression among mouse strains surveyed. (DOCX) [file pgen.1004068.s003.docx]

| **Table S1. RT-qPCR fails identify transcriptional alterations in tail skin *Col17a1* expression among mouse strains surveyed.** | | | | | | | | | | | | | | | | | | |
| --- | --- | --- | --- | --- | --- | --- | --- | --- | --- | --- | --- | --- | --- | --- | --- | --- | --- | --- |
| **A – Raw ct values of amplicons across transcript*** | | | | | | | | | | | | | | | | | | |
|  | | | *------------------------------------------------------------------****Col17a1*** -------------------------------------------------------------------- | | | | | | | | | | | | | |  | |
| **Strain** | # of Mice | | Ex1F-5R | Ex5F-10R | Ex18F-23R | Ex24F-31R | Ex31F-37R | | Ex40F-44R | Ex45F-47R | Ex47F-50R | Ex50F-53R | | Ex50F-53R | Ex51F-54R | | 18s | B2m |
| B6 | | 6 | 25.54 | 24.85 | 27.35 | 27.57 | 26.02 | | 30.01 | 26.23 | 27.37 | 27.76 | | 26.98 | 27.27 | | 15.23 | 28.47 |
| B6.chr19^PWD^ | | 5 | 25.92 | 25.27 | 26.89 | 27.56 | 26.25 | | 29.26 | 25.44 | 28.20 | 26.86 | | 27.13 | 27.37 | | 15.04 | 27.95 |
| B6-*jeb/jeb* | | 6 | 23.95 | 25.15 | 27.06 | 27.60 | 26.68 | | 29.15 | 25.57 | 27.30 | 26.81 | | 26.89 | 27.51 | | 15.06 | 28.85 |
| B6.*chr19^AJ^-jeb/jeb* | | 3 | 24.93 | 24.91 | 26.52 | 27.41 | 25.49 | | 28.81 | 25.05 | 27.36 | 26.68 | | 26.97 | 27.34 | | 14.75 | 28.14 |
| B6.*chr19^PWD^-jeb/jeb* | | 4 | 24.80 | 24.53 | 26.77 | 26.98 | 25.77 | | 28.60 | 25.28 | 26.47 | 26.34 | | 26.11 | 26.19 | | 14.55 | 27.95 |
| 129-*jeb/jeb* | | 6 | 24.26 | 26.36 | 26.24 | 26.65 | 24.95 | | 27.42 | 26.03 | 27.12 | 25.74 | | 26.31 | 26.74 | | 15.20 | 28.90 |
| DBA-*jeb/jeb* | | 5 | 22.37 | 24.88 | 26.90 | 26.59 | 24.97 | | 27.71 | 25.09 | 26.31 | 25.29 | | 26.09 | 26.83 | | 14.64 | 27.35 |
| FVB-*jeb/jeb* | | 7 | 22.23 | 24.58 | 25.50 | 26.24 | 25.01 | | 27.48 | 25.03 | 26.36 | 25.67 | | 26.21 | 27.58 | | 14.32 | 27.48 |
| MRL-*jeb/jeb* | | 6 | 21.38 | 24.29 | 25.82 | 25.99 | 24.53 | | 27.64 | 26.27 | 26.69 | 27.03 | | 26.92 | 27.31 | | 14.26 | 29.11 |
|  | | | | | | | | | | | | | | | | | | |
| **B – Oligonucleotide primers used** | | | | | | | | | | | | | | | | | | |
| **Amplicon** | | | **Forward** | | | | | **Reverse** | | | | | **cDNA**  **Size#** | | | **Genomic**  **Size#** | | |
| Col17a1 Ex1F-ex5R | | | AGCTCCTCCAGACGCGAAG | | | | | GGTAGAGGCCGGTGAGTGC | | | | | 301 | | | 10391 | | |
| Col17a1 Ex5F-ex10R | | | ATGACCCGCCATGGAACTTA | | | | | TGCTGAATCATAGGTTCCGGAC | | | | | 324 | | | 5831 | | |
| Col17a1 Ex18F-ex23R | | | CTGGATTAGGCAAGGCTGAGC | | | | | CCACGAGGTCCCATGGG | | | | | 301 | | | 2687 | | |
| Col17a1 Ex24F-ex31R | | | AGGGCCTTCCTGGTGTCC | | | | | CATCCCTGTCAAGCCTTGTTC | | | | | 323 | | | 4170 | | |
| Col17a1 Ex31F-ex37R | | | TGGAGACCCAGGAAAGCCA | | | | | GAGGACCTCAGAGATGGAGCTG | | | | | 307 | | | 5605 | | |
| Col17a1 Ex40F-ex44R | | | CAGGATCCTTCCTGACTGACTCA | | | | | GATGGTGATGACAGGCCCTG | | | | | 338 | | | 1994 | | |
| Col17a1 Ex45F-ex47R | | | TACGGTGCTGGCTTGTCCTC | | | | | GGATGTTCCTGGCAGGCC | | | | | 261 | | | 984 | | |
| Col17a1 Ex47F-ex50R | | | TACGAGGAGCTACTGACCATGC | | | | | GGCCAACGATGAAGCTGC | | | | | 316 | | | 1224 | | |
| Col17a1 Ex50F-ex53R | | | ACAACAAGCTGGCAGTGCG | | | | | TTCTCTCCTTTGTTCCCTCTGG | | | | | 301 | | | 1159 | | |
| Col17a1 Ex51F-ex54R | | | CAGGGTCTGCTACAAGGGATG | | | | | TCTTCCTTCTCCCAGTATAGACTTGG | | | | | 301 | | | 977 | | |
| B2m | | | TGCTACTCGGCGCTTCAGTC | | | | | ACAGGCCGGTCAGTGAGACA | | | | | 119 | | | 119 | | |
| 18S | | | CCGCAGCTAGGAATAATGGAAT | | | | | CGAACCTCCGACTTTCGTTCT | | | | | - | | | - | | |
| *Average ct values from the indicated number of biological replicates run in duplicate.  #Expected sizes of cDNA amplicons are indicated. Genomic DNA amplicons of larger size, which would not be efficiently amplified if genomic DNA contamination was present, are also shown. | | | | | | | | | | | | | | | | | | |
