# Supplementary material for: Molecular Identification of Collagen 17a1 as a Major Genetic Modifier of Laminin Gamma 2 Mutation-Induced Junctional Epidermolysis Bullosa in Mice
Source: PLoS Genet. 2014 Feb 13;10(2):e1004068. doi: 10.1371/journal.pgen.1004068 (PMC3923665; doi:10.1371/journal.pgen.1004068)
Supplement: Table S2 — Strain relationships of the genomic region including Col17a1 among relevant mouse strains. (DOCX) [file pgen.1004068.s004.docx]

| **Table S2.**  **Relationships of genomic regions including *Col17a1* among relevant mouse strains*.** | | | | |  | |  | | | |  | | |  | |
| --- | --- | --- | --- | --- | --- | --- | --- | --- | --- | --- | --- | --- | --- | --- | --- |
|  |  |  |  |  |  | |  | | | |  | | |  | |
|  |  |  |  |  |  | |  | | | |  | | |  | |
| Test | Compared | Chr 19 | Chr 19 | *Col17a1* |  | | |  | |  | | |  |  |  |
| Strain: | with: | 42-52 Mb | 47-48 Mb | ~47.6 Mb |  | | |  | |  | | |  |  |  |
| 129X1 | B6 | 2/2395 | 0/271 | 0/9 |  | |  | | | |  | | |  | |
|  | 129S1 | 752/2403 | 113/272 | 3/9 |  | |  | | | |  | | |  | |
|  | DBA/2 | 666/2411 | 113/272 | 3/9 |  | |  | | | |  | | |  | |
|  | PWK | 1074/2286 | 107/260 | 4/9 |  | |  | | | |  | | |  | |
| DBA/1 | B6 | 698/2385 | 114/271 | 4/10 |  | |  | | | |  | | |  | |
|  | 129S1 | 520/2395 | 28/272 | 0/10 |  | |  | | | |  | | |  | |
|  | DBA/2 | 83/2403 | 0/272 | 0/10 |  | |  | | | |  | | |  | |
|  | PWK | 1178/2280 | 133/260 | 5/10 |  | |  | | | |  | | |  | |
| PWD | B6 | 1137/2309 | 115/267 | 5/10 |  | |  | | | |  | | |  | |
|  | 129S1 | 1190/2320 | 143/268 | 5/10 |  | |  | | | |  | | |  | |
|  | DBA/2 | 1223/2326 | 144/268 | 5/10 |  | |  | | | |  | | |  | |
|  | PWK | 165/2262 | 18/257 | 0/10 |  | |  | | | |  | | |  | |
| * The number of polymorphic SNPs for the indicated strain comparisons and genetic intervals /total SNP number based on MGD CGD-MDA1 (546,000^+^ SNPs). Low polymorphism ratios for 129X1 vs B6, DBA/1 vs DBA/2 and PWD vs PWK are evidence for shared strain heritages and the likelihood that the compared strains have few polymorphisms across the entire *Col17a1* gene. | | | | | |  | | |  | | |  | | |  |
|  |  |  |  |  |  |  |  |  |  |  |  |  |  |  |  |
