# Supplementary material for: Molecular Identification of Collagen 17a1 as a Major Genetic Modifier of Laminin Gamma 2 Mutation-Induced Junctional Epidermolysis Bullosa in Mice
Source: PLoS Genet. 2014 Feb 13;10(2):e1004068. doi: 10.1371/journal.pgen.1004068 (PMC3923665; doi:10.1371/journal.pgen.1004068)
Supplement: Table S4 — Amino acid polymorphisms of human Collagen XVII based on the EVS and 1000 genome databases. (DOCX) [file pgen.1004068.s006.docx]

| **Table S4. AA variation of human Collagen XVII based on Exome Variant Server and 1000 Genomes Databases.** | | | | | | | | | | | | | | | | | | | | | | | | | | | | | |  | | | | |  |  |  |  |  |  |  |  |  |  |  |  |  |  |  |  |  |  |  |  |
| --- | --- | --- | --- | --- | --- | --- | --- | --- | --- | --- | --- | --- | --- | --- | --- | --- | --- | --- | --- | --- | --- | --- | --- | --- | --- | --- | --- | --- | --- | --- | --- | --- | --- | --- | --- | --- | --- | --- | --- | --- | --- | --- | --- | --- | --- | --- | --- | --- | --- | --- | --- | --- | --- | --- |
| **A - Variation based on Exon** | | | | | |  | **B - Variation based on protein domains** | | | | | | | | | | | **C - Variation based on AA residue** | | | | | | | | | | | | |  | | | | |  |  |  |  |  |  |  |  |  |  |  |  |  |  |  |  |  |  |  |
| Exon # | bp | # AA Exon | Variant count | | Variants /Exon size |  | Domain | | % of Protein | | # AA | | Variant count | | Variants /Domain size | | |  | | Amino acid | | Count | | Poly AA count | | % AA | | % Variant AA | | |  | | | | |  |  |  |  |  |  |  |  |  |  |  |  |  |  |  |  |  |  |  |
| 2 | 52 | 17 | 3 | | 0.18 |  | NC1 | | 1.0 | | 15 | | 3 | | 0.20 | | |  | | A | | 85 | | 14 | | 5.7 | | 7.2 | | |  | | | | |  |  |  |  |  |  |  |  |  |  |  |  |  |  |  |  |  |  |  |
| 3 | 45 | 15 | 2 | | 0.13 |  | COL1 | | 3.0 | | 45 | | 8 | | 0.18 | | |  | | C | | 7 | | 0 | | 0.5 | | 0.0 | | |  | | | | |  |  |  |  |  |  |  |  |  |  |  |  |  |  |  |  |  |  |  |
| 4 | 105 | 35 | 4 | | 0.11 |  | NC2 | | 1.6 | | 24 | | 4 | | 0.17 | | |  | | D | | 54 | | 7 | | 3.6 | | 3.6 | | |  | | | | |  |  |  |  |  |  |  |  |  |  |  |  |  |  |  |  |  |  |  |
| 5 | 129 | 43 | 8 | | 0.19 |  | COL2 | | 1.0 | | 15 | | 1 | | 0.07 | | |  | | E | | 63 | | 6 | | 4.2 | | 3.1 | | |  | | | | |  |  |  |  |  |  |  |  |  |  |  |  |  |  |  |  |  |  |  |
| 6 | 48 | 16 | 2 | | 0.13 |  | NC3 | | 3.2 | | 48 | | 6 | | 0.13 | | |  | | F | | 25 | | 1 | | 1.7 | | 0.5 | | |  | | | | |  |  |  |  |  |  |  |  |  |  |  |  |  |  |  |  |  |  |  |
| 7* | 36 | 12 | 4 | | 0.33 |  | COL3 | | 2.3 | | 35 | | 6 | | 0.17 | | |  | | G* | | 282 | | 32 | | 18.8 | | 16.4 | | |  | | | | |  |  |  |  |  |  |  |  |  |  |  |  |  |  |  |  |  |  |  |
| 8 | 48 | 16 | 0 | | 0.00 |  | NC4 | | 2.4 | | 36 | | 9 | | 0.25 | | |  | | H | | 23 | | 3 | | 1.5 | | 1.5 | | |  | | | | |  |  |  |  |  |  |  |  |  |  |  |  |  |  |  |  |  |  |  |
| 9 | 144 | 48 | 3 | | 0.06 |  | COL4 | | 1.0 | | 15 | | 1 | | 0.07 | | |  | | I | | 41 | | 6 | | 2.7 | | 3.1 | | |  | | | | |  |  |  |  |  |  |  |  |  |  |  |  |  |  |  |  |  |  |  |
| 10 | 159 | 53 | 4 | | 0.08 |  | NC5* | | 1.9 | | 29 | | 8 | | 0.28 | | |  | | K | | 60 | | 3 | | 4.0 | | 1.5 | | |  | | | | |  |  |  |  |  |  |  |  |  |  |  |  |  |  |  |  |  |  |  |
| 11 | 72 | 24 | 4 | | 0.17 |  | COL5 | | 1.4 | | 21 | | 1 | | 0.05 | | |  | | L | | 107 | | 11 | | 7.1 | | 5.6 | | |  | | | | |  |  |  |  |  |  |  |  |  |  |  |  |  |  |  |  |  |  |  |
| 12 | 72 | 24 | 2 | | 0.08 |  | NC6 | | 1.5 | | 22 | | 1 | | 0.05 | | |  | | M | | 33 | | 7 | | 2.2 | | 3.6 | | |  | | | | |  |  |  |  |  |  |  |  |  |  |  |  |  |  |  |  |  |  |  |
| 13 | 69 | 23 | 4 | | 0.17 |  | COL6 | | 1.2 | | 18 | | 3 | | 0.17 | | |  | | N | | 31 | | 3 | | 2.1 | | 1.5 | | |  | | | | |  |  |  |  |  |  |  |  |  |  |  |  |  |  |  |  |  |  |  |
| 14 | 162 | 54 | 6 | | 0.11 |  | NC7 | | 0.9 | | 14 | | 1 | | 0.07 | | |  | | P# | | 204 | | 30 | | 13.6 | | 15.4 | | |  | | | | |  |  |  |  |  |  |  |  |  |  |  |  |  |  |  |  |  |  |  |
| 15 | 81 | 27 | 4 | | 0.15 |  | COL7 | | 1.0 | | 15 | | 3 | | 0.20 | | |  | | Q | | 48 | | 2 | | 3.2 | | 1.0 | | |  | | | | |  |  |  |  |  |  |  |  |  |  |  |  |  |  |  |  |  |  |  |
| 16 | 45 | 15 | 1 | | 0.07 |  | NC8 | | 1.7 | | 25 | | 1 | | 0.04 | | |  | | R§ | | 68 | | 32 | | 4.5 | | 16.4 | | |  | | | | |  |  |  |  |  |  |  |  |  |  |  |  |  |  |  |  |  |  |  |
| 17 | 198 | 66 | 11 | | 0.17 |  | COL8 | | 1.0 | | 15 | | 1 | | 0.07 | | |  | | S | | 178 | | 15 | | 11.9 | | 7.7 | | |  | | | | |  |  |  |  |  |  |  |  |  |  |  |  |  |  |  |  |  |  |  |
| 18 | 222 | 74 | 10 | | 0.14 |  | NC9 | | 3.9 | | 58 | | 7 | | 0.12 | | |  | | T | | 84 | | 11 | | 5.6 | | 5.6 | | |  | | | | |  |  |  |  |  |  |  |  |  |  |  |  |  |  |  |  |  |  |  |
| 19 | 30 | 10 | 2 | | 0.20 |  | COL9 | | 1.0 | | 15 | | 2 | | 0.13 | | |  | | V | | 58 | | 8 | | 3.9 | | 4.1 | | |  | | | | |  |  |  |  |  |  |  |  |  |  |  |  |  |  |  |  |  |  |  |
| 20 | 27 | 9 | 1 | | 0.11 |  | NC10 | | 1.7 | | 25 | | 3 | | 0.12 | | |  | | W | | 10 | | 0 | | 0.7 | | 0.0 | | |  | | | | |  |  |  |  |  |  |  |  |  |  |  |  |  |  |  |  |  |  |  |
| 21 | 27 | 9 | 0 | | 0.00 |  | COL10 | | 1.0 | | 15 | | 2 | | 0.13 | | |  | | Y | | 36 | | 4 | | 2.4 | | 2.1 | | |  | | | | |  |  |  |  |  |  |  |  |  |  |  |  |  |  |  |  |  |  |  |
| 22 | 63 | 21 | 5 | | 0.24 |  | NC11 | | 1.0 | | 15 | | 1 | | 0.07 | | |  | | Total | | 1497 | | 195 | | 100 | | 100 | | |  | | | | |  |  |  |  |  |  |  |  |  |  |  |  |  |  |  |  |  |  |  |
| 23 | 105 | 35 | 2 | | 0.06 |  | COL11 | | 1.8 | | 27 | | 0 | | 0.00 | | |  | | * COL domains, including GXY triplet motifs, make up | | | | | | | | | | | |  | | | | | | | | | |  |  |  |  |  |  |  |  |  |  |  |  |  |
| 24 | 63 | 21 | 1 | | 0.05 |  | NC12 | | 0.8 | | 12 | | 3 | | 0.25 | | |  | | Only 6% of the substitutions for G map outside of COL domains. | | | | | | | | | | | | | | | |  | | | |  | | | | |  | | |  | | | |  | | |
|  |  |  |  |  |  |  |  |  |  |  |  |  |  |  |  |  |  |  |  | 39% of the protein. Only 6% of the substitutions for | | | | | | | | | | | | | |  | | | |  | | | | |  | | | |  | | | |  | | |  |
| 25 | 36 | 12 | 2 | | 0.17 |  | COL12 | | 2.2 | | 33 | | 7 | | 0.21 | | |  | | G map outside of COL domains. | | | | | | | | | | | | | | | |  | | | |  | | | | |  | | |  | | | |  | | |
| 26 | 54 | 18 | 1 | | 0.06 |  | NC13 | | 0.6 | | 9 | | 2 | | 0.22 | | |  | | # COL domains include GPP or GPY triplets. Of the 30 | | | | | | | | | | | | | | | |  | | | |  | | | | |  | | |  | | | |  | | |
| 27 | 36 | 12 | 1 | | 0.08 |  | COL13* | | 2.7 | | 40 | | 8 | | 0.20 | | |  | | substitutions for P, 26 are in COL segments. | | | | | | | | | | | | | | | |  |  |  |  |  |  |  |  |  |  |  |  |  |  |  |  |  |  |  |
| 28 | 36 | 12 | 1 | | 0.08 |  | NC14 | | 0.4 | | 6 | | 0 | | 0.00 | | |  | |  | |  | |  | |  | |  | | |  | | | | |  |  |  |  |  |  |  |  |  |  |  |  |  |  |  |  |  |  |  |
| 29 | 63 | 21 | 0 | | 0.00 |  | COL14 | | 2.0 | | 30 | | 2 | | 0.07 | | |  | |  | |  | |  | |  | |  | | |  | | | | |  |  |  |  |  |  |  |  |  |  |  |  |  |  |  |  |  |  |  |
| 30 | 36 | 12 | 0 | | 0.00 |  | NC15 | | 0.8 | | 12 | | 2 | | 0.17 | | |  | |  | |  | |  | |  | |  | | |  | | | | |  |  |  |  |  |  |  |  |  |  |  |  |  |  |  |  |  |  |  |
| 31 | 72 | 24 | 3 | | 0.13 |  | COL15 | | 16.2 | | 242 | | 26 | | 0.11 | | |  | |  | |  | |  | |  | |  | | |  | | | | |  |  |  |  |  |  |  |  |  |  |  |  |  |  |  |  |  |  |  |
| 32 | 27 | 9 | 1 | | 0.11 |  | NC16 | | 5.1 | | 77 | | 11 | | 0.14 | | |  | |  | |  | |  | |  | |  | | |  | | | | |  |  |  |  |  |  |  |  |  |  |  |  |  |  |  |  |  |  |  |
| 33* | 36 | 12 | 5 | | 0.42 |  | TM | | 1.5 | | 23 | | 0 | | 0.00 | | |  | |  | |  | |  | |  | |  | | |  | | | | |  |  |  |  |  |  |  |  |  |  |  |  |  |  |  |  |  |  |  |
| 34 | 36 | 12 | 3 | | 0.25 |  | IC | | 31.1 | | 466 | | 62 | | 0.13 | | |  | |  | |  | |  | |  | |  | | |  | | | | |  |  |  |  |  |  |  |  |  |  |  |  |  |  |  |  |  |  |  |
| 35 | 81 | 27 | 3 | | 0.11 |  | Total | | 100 | | 1497 | | 195 | | x =0.13 | | |  | |  | |  | |  | |  | |  | | |  | | | | |  |  |  |  |  |  |  |  |  |  |  |  |  |  |  |  |  |  |  |
| 36 | 36 | 12 | 0 | | 0.00 |  |  | |  | |  | |  | | sd=0.076 | | |  | |  | |  | |  | |  | |  | | |  | | | | |  |  |  |  |  |  |  |  |  |  |  |  |  |  |  |  |  |  |  |
| 37 | 54 | 18 | 4 | | 0.22 |  | * NC5 exceeds variant frequency/domain size by | | | | | | | | | | |  | |  | |  | |  | |  | |  | | |  | | | | |  |  |  |  |  |  |  |  |  |  |  |  |  |  |  |  |  |  |  |
| 38 | 42 | 14 | 4 | | 0.29 |  | ≥2 SD. | | | | | | | | | | | | | | | | | | | | |  | | |  | | | | |  |  | |  | |  | | |  | |  | | |  |  | | |  | |
| 39 | 54 | 18 | 0 | | 0.00 |  |  |  | | |  | |  | |  | | | |  |  | |  | |  | |  | |  | | |  | | | | |  |  |  |  |  |  |  |  |  |  |  |  |  |  |  |  |  |  |  |
| 40 | 60 | 20 | 5 | | 0.25 |  |  |  | | |  | |  | |  | | | |  |  | |  | |  | |  | |  | | |  | | | | |  |  |  |  |  |  |  |  |  |  |  |  |  |  |  |  |  |  |  |
| 41 | 27 | 9 | 2 | | 0.22 |  |  |  | | |  | |  | |  | | | |  |  | |  | |  | |  | |  | | |  | | | | |  |  |  |  |  |  |  |  |  |  |  |  |  |  |  |  |  |  |  |
| 42 | 33 | 11 | 2 | | 0.18 |  |  |  | | |  | |  | |  | | | |  |  | |  | |  | |  | |  | | |  | | | | |  |  |  |  |  |  |  |  |  |  |  |  |  |  |  |  |  |  |  |
| 43 | 75 | 25 | 3 | | 0.12 |  |  |  | | |  | |  | |  | | | |  |  | |  | |  | |  | |  | | |  | | | | |  |  |  |  |  |  |  |  |  |  |  |  |  |  |  |  |  |  |  |
| 44 | 51 | 17 | 0 | | 0.00 |  |  |  | | |  | |  | |  | | | |  |  | |  | |  | |  | |  | | |  | | | | |  |  |  |  |  |  |  |  |  |  |  |  |  |  |  |  |  |  |  |
| 45 | 123 | 41 | 4 | | 0.10 |  |  |  | | |  | |  | |  | | | |  |  | |  | |  | |  | |  | | |  | | | | |  |  |  |  |  |  |  |  |  |  |  |  |  |  |  |  |  |  |  |
| 46 | 138 | 46 | 8 | | 0.17 |  |  |  | | |  | |  | |  | | | |  |  | |  | |  | |  | |  | | |  | | | | |  |  |  |  |  |  |  |  |  |  |  |  |  |  |  |  |  |  |  |
| 47 | 69 | 23 | 2 | | 0.09 |  |  |  | | |  | |  | |  | | | |  |  | |  | |  | |  | |  | | |  | | | | |  |  |  |  |  |  |  |  |  |  |  |  |  |  |  |  |  |  |  |
| 48 | 141 | 47 | 3 | | 0.06 |  |  |  | | |  | |  | |  | | | |  |  | |  | |  | |  | |  | | |  | | | | |  |  |  |  |  |  |  |  |  |  |  |  |  |  |  |  |  |  |  |
| 49 | 90 | 30 | 3 | | 0.10 |  |  |  | | |  | |  | |  | | | |  |  | |  | |  | |  | |  | | |  | | | | |  |  |  |  |  |  |  |  |  |  |  |  |  |  |  |  |  |  |  |
| 50 | 111 | 37 | 5 | | 0.14 |  |  |  | | |  | |  | |  | | | |  |  | |  | |  | |  | |  | | |  | | | | |  |  |  |  |  |  |  |  |  |  |  |  |  |  |  |  |  |  |  |
| 51 | 147 | 49 | 7 | | 0.14 |  |  |  | | |  | |  | |  | | | |  |  | |  | |  | |  | |  | | |  | | | | |  |  |  |  |  |  |  |  |  |  |  |  |  |  |  |  |  |  |  |
| 52 | 390 | 130 | 22 | | 0.17 |  |  |  | | |  | |  | |  | | | |  |  | |  | |  | |  | |  | | |  | | | | |  |  |  |  |  |  |  |  |  |  |  |  |  |  |  |  |  |  |  |
| 53 | 138 | 46 | 6 | | 0.13 |  |  |  | | |  | |  | |  | | | |  |  | |  | |  | |  | |  | | |  | | | | |  |  |  |  |  |  |  |  |  |  |  |  |  |  |  |  |  |  |  |
| 54 | 63 | 21 | 2 | | 0.10 |  |  |  | | |  | |  | |  | | | |  |  | |  | |  | |  | |  | | |  | | | | |  |  |  |  |  |  |  |  |  |  |  |  |  |  |  |  |  |  |  |
| 55 | 81 | 27 | 7 | | 0.26 |  |  |  | | |  | |  | |  | | | |  |  | |  | |  | |  | |  | | |  | | | | |  |  |  |  |  |  |  |  |  |  |  |  |  |  |  |  |  |  |  |
| 56 | 55 | 18 | 3 | | 0.16 |  |  |  | | |  | |  | |  | | | |  |  | |  | |  | |  | |  | | |  | | | | |  |  |  |  |  |  |  |  |  |  |  |  |  |  |  |  |  |  |  |
| Total: | 4493 | 1497 | | 195 | x=0.13 |  |  |  | | |  | |  | |  | | | |  |  | |  | |  | |  | |  | | |  | | | | |  |  |  |  |  |  |  |  |  |  |  |  |  |  |  |  |  |  |  |
|  |  |  | |  | sd=0.09 |  |  |  |  |  |  |  |  |  |  |  |  |  |  |  |  |  |  |  |  |  |  |  |  |  |  |  |  |  |  |  |  |  |  |  |  |  |  |  |  |  |  |  |  |  |  |  |  |  |
| * Exons 7 and 33 exceed variant frequency/exon size by ≥2 SD. | | | | | |  | | | |  | |  | |  | |  |  | | | |  | |  | |  | |  | |  | | | |  |  |  |  |  |  |  |  |  |  |  |  |  |  |  |  |  |  |  |  |  |  |
