# Supplementary material for: Molecular Identification of Collagen 17a1 as a Major Genetic Modifier of Laminin Gamma 2 Mutation-Induced Junctional Epidermolysis Bullosa in Mice
Source: PLoS Genet. 2014 Feb 13;10(2):e1004068. doi: 10.1371/journal.pgen.1004068 (PMC3923665; doi:10.1371/journal.pgen.1004068)
Supplement: Table S5 — Localization of a PRDM9 binding site in the 1085 bp recombinant interval of Col17a1. (DOCX) [file pgen.1004068.s007.docx]

**Table S5. Localization of a PRDM9 binding site in the 1085 bp recombinant interval of *Col17a1.***

TTTCAATCCCCACCCATCTGATTCCAGAGCTGCTATCTACACAGTCCAAGTACATAAAAAAACGAGGGCCAGTTTTAGGCCTGCAAAGCCTAAATGCTGAGGTCCACACATGCTCCTTCCCCCTCTGAGGGCCACAGGTTCCAACTTACGCTGCATGCTCTCCGACACCCGCACTGCCAGCTTGTTGTAATCTAGGTCTCCAGTGAAGCCATCCCGGAACGAGTCACCATTGGTGCCATATATACCACCACCTGCAGCTGCCCCATAGCCTCCGCCTGGGCCGATGTCGGTACCATAGGGGCCCCCATCTCCTGCACCATAGGCTCCACCTTCACCCAGGGAGCCTCCGTTGGTTCCCCCTGTGCCCGTGGAAGAGCTGTAGGAAGTGCCCCTTCTGGCTGAGGAGTTGCTGCTCCAGTTGTAGTTCTCCCTTAAGTGACCATCTCCAGGTGGTCCCTGTGGCCCCGGAGGGCCTGGAGGGCCAACGATGAAGCTGCGAACATCAGGACCTGTAGGGGAGGAAGATGCATTACCGGCAGACTGTGGTGGACAAAAGAGTTGGGAGCTAGGGGGGTCTCTGGAGTTTTAACACCCCTTAGAGTGACCTTTGAGGCCCTTGTACATGTACAGGATGCTGGTGAGCAGAGAGTGGTTTATCTAGACTGCTGACCATCCTGTGGCTCCTGCAGAAGAGTTGTGAAGGTCTAGGGGGAGGGCACAGGAGACCAACATCAAGGTACCTACTTGTGAGGTAGCTAATCAGCTCGCTGCGGAAGTTGTCGCTGTTCTCAGCTGCATAGGTGGACAGAGCTGCTGAGACACCTGGGGGCCCTCGAGGACCTGGGGGCCCTGGTGGGCCTGGAGGGCCTGGGATGGAGGACAAACCAGCGGCTGTGTGAGAGGGGAGGGAAAGAGGAAGGTCAGGATCCTGGACAGAAAAGGCTAGATTCTCAGCCTGGCCTTGACCTACTGCGGGCCCCCTCACCCCCCATGGATTGGGTGCAAGAGATGGGTATGTGTGTGTGTGTGTGTGTGGAGTAGAGGAGAGTGGGCAAGGCCTAGGTTGTCCCTTTCAGGCGATATCT

19:47648582 – 47649666 Build 38

Blue, the most probable PRDM9 binding site according to the Persikov – Singh algorithm [[32](#_ENREF_32)].

Larger font, matches to the most frequent bases in the *Prdm9^Dom2^* (B6 allele) motif (Baker CL, Walker M, Kajita S, Petkov PM, Paigen K. PRDM9 binding organizes hotspot nucleosomes and limits Holliday junction migration, *submitted*.)
